# The Effectiveness of Strategies to Improve User Engagement With Digital Health Interventions Targeting Nutrition, Physical Activity, and Overweight and Obesity: Systematic Review and Meta-Analysis

**DOI:** 10.2196/47987

**Published:** 2023-12-19

**Authors:** Alice Grady, Nicole Pearson, Hannah Lamont, Lucy Leigh, Luke Wolfenden, Courtney Barnes, Rebecca Wyse, Meghan Finch, Matthew Mclaughlin, Tessa Delaney, Rachel Sutherland, Rebecca Hodder, Sze Lin Yoong

**Affiliations:** 1 School of Medicine and Public Health University of Newcastle Callaghan Australia; 2 Hunter New England Population Health Hunter New England Local Health District Wallsend Australia; 3 Population Health Research Program Hunter Medical Research Institute New Lambton Australia; 4 National Centre of Implementation Science University of Newcastle Callaghan Australia; 5 College of Health, Medicine and Wellbeing University of Newcastle Callaghan Australia; 6 Data Sciences Hunter Medical Research Institute New Lambton Australia; 7 Equity in Health and Wellbeing Program Hunter Medical Research Institute New Lambton Australia; 8 Telethon Kids Institute University of Western Australia Perth Australia; 9 Global Obesity Centre, Institute for Health Transformation School of Health and Social Development Deakin University Melbourne Australia

**Keywords:** engagement, digital health interventions, systematic review, nutrition, physical activity, obesity

## Abstract

**Background:**

Digital health interventions (DHIs) are effective in improving poor nutrition, physical inactivity, overweight and obesity. There is evidence suggesting that the impact of DHIs may be enhanced by improving user engagement. However, little is known about the overall effectiveness of strategies on engagement with DHIs.

**Objective:**

This study aims to assess the overall effectiveness of strategies to improve engagement with DHIs targeting nutrition, physical activity, and overweight or obesity and explore associations between strategies and engagement outcomes. The secondary aim was to explore the impact of these strategies on health risk outcomes.

**Methods:**

The MEDLINE, Embase, PsycINFO, CINAHL, CENTRAL, Scopus, and Academic Source Complete databases were searched up to July 24, 2023. Eligible studies were randomized controlled trials that evaluated strategies to improve engagement with DHIs and reported on outcomes related to DHI engagement (use or user experience). Strategies were classified according to behavior change techniques (BCTs) and design features (eg, supplementary *emails*). Multiple-variable meta-analyses of the primary outcomes (usage and user experience) were undertaken to assess the overall effectiveness of strategies. Meta-regressions were conducted to assess associations between strategies and use and user experience outcomes. Synthesis of secondary outcomes followed the “Synthesis Without Meta-Analysis” guidelines. The methodological quality and evidence was assessed using the Cochrane risk-of-bias tool, and the Grading of Recommendations Assessment, Development, and Evaluation tool respectively.

**Results:**

Overall, 54 studies (across 62 publications) were included. Pooled analysis found very low-certainty evidence of a small-to-moderate positive effect of the use of strategies to improve DHI use (standardized mean difference=0.33, 95% CI 0.20-0.46; *P*<.001) and very low-certainty evidence of a small-to-moderate positive effect on user experience (standardized mean difference=0.29, 95% CI 0.07-0.52; *P*=.01). A significant positive association was found between the BCTs *social support* (effect size [ES]=0.40, 95% CI 0.14-0.66; *P*<.001) and *shaping knowledge* (ES=0.39, 95% CI 0.03-0.74; *P*=.03) and DHI use. A significant positive association was found among the BCTs *social support* (ES=0.70, 95% CI 0.18-1.22; *P*=.01), *repetition and substitution* (ES=0.29, 95% CI 0.05-0.53; *P*=.03), and *natural consequences* (ES=0.29, 95% CI 0.05-0.53; *P*=.02); the design features *email* (ES=0.29, 95% CI 0.05-0.53; *P*=.02) and SMS text messages (ES=0.34, 95% CI 0.11-0.57; *P*=.01); and DHI user experience. For secondary outcomes, 47% (7/15) of nutrition-related, 73% (24/33) of physical activity–related, and 41% (14/34) of overweight- and obesity-related outcomes reported an improvement in health outcomes.

**Conclusions:**

Although findings suggest that the use of strategies may improve engagement with DHIs targeting such health outcomes, the true effect is unknown because of the low quality of evidence. Future research exploring whether specific forms of social support, repetition and substitution, natural consequences, emails, and SMS text messages have a greater impact on DHI engagement is warranted.

**Trial Registration:**

PROSPERO CRD42018077333; https://www.crd.york.ac.uk/prospero/display_record.php?RecordID=77333

## Introduction

### Burden of Chronic Disease

Chronic diseases such as cancer, cardiovascular disease, and type 2 diabetes account for 73% of deaths and 61% of all disability-adjusted life years worldwide [1,2]. Overweight and obesity, poor nutrition, and physical inactivity are among the leading modifiable risk factors for chronic diseases, and the development of interventions to address these risk factors has been identified as a public health priority internationally [3]. Interventions that target overweight and obesity, nutrition, and physical activity need to be scalable and low cost to have a wide-reaching impact on the prevalence of chronic diseases [4].

### Digital Health Interventions Can Improve Chronic Disease Health Risks

The use of digital health interventions (DHIs) shows promise and has been recognized by the World Health Organization (WHO) as a way to successfully deliver health interventions and reduce risk factors for chronic diseases at a population level [5-7]. Digital health describes the general use of information and communications technologies for health and is inclusive of both mobile health and eHealth, for example, websites, mobile phone communication, and apps [8]. With >5 billion internet users worldwide, these technologies have the potential to deliver effective health interventions at scale with high fidelity and at a low cost in addition to addressing health inequities by enabling the delivery of sophisticated services to individuals and communities that find traditional forms of services inaccessible [9,10]. Systematic reviews of research trials evaluating the use and effectiveness of DHIs have provided modest evidence that such interventions can improve nutrition [4,11,12], physical activity [13], and weight status [4,14-16]. However, various challenges to optimizing the effectiveness of DHIs for the prevention of chronic diseases have been identified, in particular a lack of initial and sustained user engagement [17-19].

### The Impact of DHIs Is Often Impeded by a Lack of User Engagement

In total, 2 recently published conceptual models of digital engagement have proposed multifaceted definitions of digital engagement that include both the extent to which a DHI is *used* (eg, frequency, amount and duration of access, and depth and completion of the program) and *user experience* (characterized by factors such as attention, interest, and affect) [20,21]. The reporting of both types of engagement outcomes has been recommended to provide a comprehensive understanding of DHI engagement [22]. However, to date, most studies examining DHI engagement have reported only on measures of use (eg, number of log-ins or digital activities completed as intended by the developer) [22]. Such studies frequently report that DHIs are often not adopted and used as intended [17], with a systematic review of DHIs reporting “use as intended” as being as low as 50% in research trials [23] and continued use of health apps after 30 days as low as 4% outside of a “trial context” [24]. Such findings are concerning as there is evidence suggesting that the health impact of DHIs may be enhanced by greater engagement; for example, a review by Donkin et al [25] of 33 DHI studies found that the number of log-ins was associated with an improvement in health behavior outcomes, including fruit and vegetable intake, weight status, smoking, and physical activity. More recently, a systematic review of DHIs targeting physical activity found a significant positive relationship between physical activity and user engagement (subjective experience, activities completed, and log-ins) [26], and another review of DHIs targeting nutrition found early evidence of an association between DHI use and dietary intake [27].

### How Might Strategies Improve Engagement With DHIs?

Conceptual models of engagement with DHIs suggest that exposure to engagement strategies within or adjunct to a DHI leads to increased engagement (use and user experience) with the active or core components of the DHI. Increased engagement with the DHI then leads to changes in determinants of behavior (eg, capability, opportunity, and motivation) that, in turn, influence health behaviors (eg, physical activity) and health outcomes (eg, development of chronic diseases) [20,22,28]. Despite the role of engagement in improving the health outcomes of DHIs, digital health technologies do not routinely incorporate user-engaging strategies into intervention designs [29], and trials that test strategies to maximize user engagement with interventions are only beginning to emerge [20]. Therefore, research to better understand the varying strategies that may be used to improve engagement with DHIs is warranted [20,21,30].

It has been proposed that several aspects of DHIs, including the content of the DHI itself and design and delivery features of DHIs, may enhance engagement [20,21]. Previously, behavior change techniques (BCTs) have been used to classify the types of strategies used as part of a DHI design to improve engagement [31]. Michie et al [32] have developed a taxonomy of BCTs, providing standardized labels and definitions and allowing for the coding of BCTs used in interventions. The use of BCTs may improve engagement via multiple mechanisms. For example, the use of the BCT *prompts and reminders* may directly increase engagement by reminding the user to log in to a website or complete an activity such as a diet or step log [31]. In addition, the BCT *provision of tailored feedback* may increase engagement by increasing perceived user relevance, which in turn motivates the user to interact more with the DHI [19]. The design features used as part of a DHI have also been identified as strategies with the potential to improve engagement [20,31]. For example, this may take the form of the use of *automated functions* that can increase interaction via videos and games or via *communicative functions* such as discussion boards or live chats [33]. Similarly, the addition of SMS text messages, emails, or phone calls used in adjunct to a web-based DHI could improve engagement by creating an additional access route to the end user, with opportunities for prompting DHI use [34] or increasing user attention or interest in the DHI [35]. Therefore, for the purpose of this review, we use the term “strategy” to cover elements of the DHI content, design, and delivery (classified using BCTs) [32] and a system developed by Webb et al [36].

### Why Is It Important to Conduct This Review?

Although multiple reviews have narratively synthesized the effect of strategies in improving engagement with DHIs [25,36-40], to our knowledge, only 1 review has undertaken a meta-analysis to quantify the effect of strategies on DHI engagement [31], just 1 review has undertaken multiple regression to explore the types of strategies associated with engagement [23], and no reviews have synthesized the effect of strategies on individual health risk outcomes targeted by the DHI. To date, the findings from these reviews suggest that the use of digital prompts may improve engagement with technology-based interventions [23,31]. However, these and other reviews examining the impact of strategies on engagement with DHIs have been limited in scope because of the exclusion of studies of interventions with nondigital supplementary strategies [31,39], the restriction of inclusion criteria by DHI types (ie, web-based interventions only) [23,37], the restriction of inclusion criteria to a single health risk (eg, substance use only) [40], and the use of narrow engagement outcome definitions (ie, use analytics only) [23,31,37,39]. In particular, reviews that have only examined use-based outcomes (eg, log-ins and time spent using the DHI) of engagement do not consider the multifaceted nature of digital engagement, which extends to the emotional and cognitive aspects of the user’s experience [22]. It has been recommended that the addition of user experience outcomes can provide an indication of how strategies may have influenced the behavior change process [22], providing a richer understanding of possible engagement mechanisms [20,21]. Given this, there is a need for a comprehensive systematic review addressing the limitations of previous reviews, using broader definitions of engagement, and identifying the types of strategies that can be used to improve DHI engagement. This review will be the first to provide insights into the effectiveness of strategies to improve engagement with DHIs targeting nutrition, physical activity, and overweight and obesity as well as explore the types of strategies associated with engagement. The application of the review findings is likely to ultimately enhance the public health impact of such interventions.

### Objectives

The primary aims of the review were to (1) assess the overall effectiveness of strategies to improve user engagement (use and user experience) with DHIs targeting nutrition, physical activity, and overweight and obesity; and (2) explore the association between individual strategies to improve engagement and engagement outcomes. The secondary aim was to explore the impact of strategies to improve engagement on health risk outcomes (nutrition, physical activity, weight status, or adiposity).

## Methods

This review was conducted in line with the *Cochrane Handbook for Systematic Reviews of Interventions* [41] and was prospectively registered with PROSPERO (CRD42018077333) [42].

### Eligibility Criteria

#### Types of Studies

Only randomized controlled trials (RCTs), cluster RCTs, quasi-RCTs, and cluster quasi-RCTs were included as they are considered the most robust and reliable designs for establishing the effectiveness of an intervention. Studies were included if they compared a strategy to improve user engagement with DHIs with no strategy (ie, control) or “usual care” or compared ≥2 strategies. Studies using other designs, including controlled before-and-after studies, interrupted time series, and multiple-baseline and observational studies, were excluded.

To be included, trials were required to report on the impact of a defined strategy on an engagement outcome between experimental groups receiving the same DHI, for example, a trial evaluating the impact of a web-based program with the addition of SMS text messages versus the same web-based program without SMS text messages on website engagement. Studies that randomized participants to different DHIs (eg, app vs website) or to a DHI versus a non-DHI (eg, app vs face-to-face) were not included.

#### Types of Participants

We included any study undertaken with adult participants (aged ≥18 y), including individuals or groups of individuals (eg, health care providers and community organizations). Studies targeting the health outcomes of children or families via parent use of DHIs were included. Studies only reporting the outcomes of children (individuals aged <18 y) were excluded.

#### Types of Interventions

Any studies evaluating the use of strategies to improve user engagement with DHIs aimed primarily at preventing chronic diseases by improving physical activity, nutrition, and overweight and obesity (or a combination of these) were included. Consistent with the WHO definition, DHIs were defined as the use of digital, mobile, electronic, and wireless technologies to support the achievement of health objectives [8]. DHIs included but were not limited to devices used to deliver the intervention, such as mobile phones, portable tablets (eg, iPad), web-based interventions, mobile apps, and activity trackers (eg, Fitbit). Strategies for improving engagement included but were not limited to BCTs (such as prompts and reminders, incentives, self-monitoring, or problem-solving) or different design features (such as supplementary SMS text messages or emails, automated functions, or communicative functions).

#### Types of Outcome Measures

##### Primary Outcomes

Studies that included any quantitative measure of DHI engagement, defined as the extent of DHI use as well as the user experience, characterized by but not limited to attention, interest, and affect, were included [20]. Examples of behavioral use outcomes include number of log-ins, frequency of use, number of activities completed, duration of access, and completion of the program. Examples of user experience outcomes include user satisfaction, acceptability, attention, and usability [22]. Similarly to previous reviews, engagement could be assessed via any objective or subjective quantitative measure, for example, via embedded data collection systems (ie, DHI analytics) or observation (eg, eye tracking) or as measured using self-reported questionnaires or surveys (eg, user satisfaction questionnaires) [26,27].

##### Secondary Outcomes

Secondary outcomes included any measure of nutrition, physical activity, weight status, or adiposity measured objectively or subjectively (eg, self-reported). In contrast to the registered protocol [42], this paper does not report on estimates of absolute costs or adverse effects because of challenges in defining and separating costs and adverse events resulting from strategies from DHIs overall.

#### Exclusion Criteria

The following exclusion criteria were applied: (1) studies that did not involve the use of a DHI; (2) studies that reported engagement with CD-ROM and computer-based interventions as these do not function in a web-based capacity (therefore not meeting the WHO definition of a DHI); (3) studies in which data related to engagement with a DHI could not be separated from data related to recruitment, participation, and retention within the research trial; (4) studies that did not have a primary or secondary aim to examine engagement (or related concepts, eg, use, acceptability, and feasibility) with a DHI; (5) studies in which the interventions were targeted at those with existing health-related conditions or diagnoses (eg, chronic diseases, communicable diseases, or mental illness) given our interest in generalizing findings to the general population; and (6) studies that did not quantitatively measure engagement with the DHI as an outcome.

### Search Methods for Identification of Studies

#### Overview

We performed a comprehensive search for published studies across a broad range of information sources to reflect the cross-disciplinary nature of the topic. Studies published in English were eligible. There were no restrictions regarding article publication date, length of the study follow-up period, or country of origin.

#### Electronic Searches

Searches for peer-reviewed literature were undertaken by an experienced librarian using the following electronic databases from inception to July 24, 2023: Cochrane Library (including CENTRAL), MEDLINE, Embase, PsycINFO, CINAHL, Scopus, and Academic Source Complete.

The MEDLINE search strategy (Multimedia Appendix 1) was adapted for each database using database-specific subject headings and filters. We included filters used in other systematic reviews for study design (RCTs) [43,44], intervention strategies [40], engagement outcomes [20,40], and DHIs [20,31,40]. This review was conducted alongside another review that aimed to assess the effectiveness of strategies to increase engagement with DHIs targeting smoking and alcohol consumption. As such, search terms to identify studies including DHIs targeting smoking and alcohol consumption were also applied [45,46]. A review of the studies including DHIs targeting smoking and alcohol consumption will be published separately.

#### Searching Other Resources

We screened the reference lists of all the included trials for citations of other potentially eligible studies. To ensure comprehensive identification of relevant studies that were inadequately indexed or not captured via the database search, we conducted hand searches of all publications over a 10year period (April 2013-July 2023) in the following journals as the leading and largest digital health journals worldwide: *Journal of Medical Internet Research*, *JMIR mHealth and uHealth*, *JMIR Medical Informatics*, and *JMIR Public Health and Surveillance*.

#### Selection of Studies

In accordance with the methods recommended in the *Cochrane Handbook for Systematic Reviews of Interventions* [41], pairs of reviewers (AG, NP, MM, MF, RW, HL, and Tonelle Handley) independently screened abstracts and titles for potentially eligible studies using the systematic review management system Covidence (Veritas Health Innovation) [47]. Duplicates were initially identified and removed in EndNote X9.2 (Clarivate Analytics) using the automated deduplication feature before this process was repeated in Covidence. We obtained the full texts of all potentially relevant or unclear articles, and pairs of reviewers (AG, NP, CB, TD, RH, HL, and Tonelle Handley) independently screened these against our inclusion criteria. Screening disagreements were resolved through discussion between the pairs of review authors and, where required, by consulting a third review author. The review authors were not blinded to author or journal information. The number of articles identified, screened, eligible, and included was recorded according to the PRISMA (Preferred Reporting Items for Systematic Reviews and Meta-Analyses) statement [48].

### Data Extraction and Management

#### Overview

Pairs of reviewers (AG, NP, HL, CB, Fiona Stacey, and Kate Reid) independently extracted information from the included studies. Those extracting data were not blinded to author or journal information. Data were extracted using a standardized tool adapted from the *Cochrane Public Health Group Methods Manual* [49] previously used by the review team in a systematic review [46]. All data extraction disagreements were resolved through discussion between the pairs of review authors and, where required, by consulting a third review author.

We attempted to contact the authors of studies with missing data to obtain the required primary outcome data for inclusion in the review. Where multiple publications of the same trial were included, we extracted data from those deemed the most applicable to the primary aim of this review. Data related to study arms that were not provided with a DHI (ie, true controls) were not extracted as they did not report on digital engagement outcomes.

The following information was extracted:

1. Study design, first author surname, year of publication, country, participant characteristics, settings, unit of allocation, unit of analysis, and information to allow for risk of bias (ROB) and certainty of evidence assessment (Grading of Recommendations Assessment, Development, and Evaluation; GRADE)
2. Characteristics of the intervention, including type of DHI, duration, number and frequency of strategies, and the theoretical underpinning of the intervention (if reported)
3. Primary and secondary outcome results (relevant to the review), including data collection method, outcome measures, follow-up period, effect size (ES), and all data required to undertake synthesis
4. Health risk outcomes (related to nutrition, physical activity, and overweight and obesity)
5. Source or sources of research funding and potential conflicts of interest

#### Coding of Intervention Characteristics

To standardize the types of strategies used to improve engagement, a coding system used in a previous review conducted by Webb et al [36], which categorized strategies into BCTs and design features, was used. Pairs of reviewers (AG, HL, NP, and Melanie Lum) independently coded intervention descriptions using the taxonomy of BCTs developed by Michie et al [32] and the design features (modes of delivery) as developed by Webb et al [36]. The BCT groupings and the design features used, along with example applications, can be found in Multimedia Appendix 2 [32,36]. When coding BCTs, the overarching 16 BCT groupings (rather than the 93 subcategories) were used to identify the specific techniques used in the relevant intervention arm or arms in the included studies. The description and examples within the subcategories were considered when categorizing strategies [32]. Consistent with previous studies, the design features used included 11 possible modes of delivery grouped according to 3 categories: automated functions, communicative functions, and use of supplementary modes [36,50]. BCTs and design features were only coded when they were identified as strategies to increase engagement in an intervention arm but not in the comparison arm. For example, if multiple BCTs were coded in both the intervention and comparison arms, only the BCTs unique to one arm were identified as the strategies to increase engagement. As there was no limit or predefined range of strategies included in the review, any unique BCTs and design features were recorded irrespective of whether the study authors indicated that these strategies were specifically designed to improve engagement.

#### Assessment of ROB in the Included Studies

Pairs of reviewers (AG, NP, RS, Sam McCrabb, Li Chai, and Tonelle Handley) independently assessed the ROB using the “risk-of-bias” tool described in the *Cochrane Handbook for Systematic Reviews of Interventions* [41]. An ROB judgment (“high,” “low,” or “unclear”) was determined for each of the domains assessed, including sequence generation, allocation concealment, blinding of participants and personnel, blinding of outcome assessment, incomplete outcome data, selective outcome reporting, and “other” potential sources of bias. Additional criteria for cluster RCTs (recruitment to cluster, baseline imbalance, loss of clusters, incorrect analysis, contamination, and compatibility with individually randomized RCTs) were also included. Separate ROB assessments were conducted on the primary outcomes (use and user experience) and for the study overall [51]. High study ROB was defined as scoring high on at least half of the domains assessed, unclear study ROB was defined as scoring unclear or low on at least half of the domains assessed, and low study ROB was defined as scoring low on at least half of the domains assessed [52]. ROB disagreements were resolved through discussion between the pairs of review authors and, where required, by consulting a third review author.

#### Unit-of-Analysis Issues

Studies using clustering were examined for unit-of-analysis errors and identified in the “Risk of bias.” For both primary outcomes, the intracluster correlation coefficient reported in the included studies was used. If the trial did not account for clustering, it was accounted for by adjusting the total sample size for the design effect [41].

#### GRADE Tool

Pairs of reviewers (AG and NP) used the GRADE system to independently assess the certainty of the body of evidence for the primary engagement outcomes by considering the study limitations, consistency of effect, imprecisions, indirectness, and publication bias [51]. GRADE disagreements were resolved through discussion between the pair of review authors and, where required, by consulting a third review author. The certainty of the body of evidence for the main engagement meta-analyses was graded from “High” to “Very-Low” in accordance with the *Cochrane Handbook for Systematic Reviews of Interventions* [41].

### Data Synthesis and Analysis

#### Meta-Analyses

We undertook 2 multivariate meta-analyses, one for outcomes measuring DHI use (eg, number of log-ins or web visits, time spent using the DHI, and number or proportion of DHI activities completed) and one for outcomes measuring user experience (eg, acceptability, satisfaction, usability, and attention) to assess the impact of strategies on each conceptual model of engagement. Given the broad definitions of both use and user experience, multivariate meta-analyses were undertaken as they allow for the joint synthesis of multiple outcomes accounting for their correlation. As this approach allows more studies to contribute to the meta-analysis, it may improve efficiency and decrease bias (because of selective outcome reporting) [53].

All DHI use and user experience outcomes of interest to the review were included in the meta-analyses. Most included studies (51/54, 94%) reported multiple measures of engagement, had multiple intervention arms, or both. Only study arms reporting on engagement outcomes compared with the “control arm” were included in the analysis. To account for multiple outcome measures, robust variance estimation (RVE) meta-analyses were used to account for the unknown correlations in each study. RVE produces valid SEs, point estimates, and CIs without needing to know the exact within-study correlations and is robust to the choice of within-study correlation [54]. For factorial and multiarm trials, the outcomes for each relevant arm were entered separately, with the correlation between the results handled by the multivariate model. All outcomes (both continuous and categorical) were converted to standardized mean differences (SMDs) and their corresponding sampling variances. When multiple time points were reported in the included studies, we chose the time point closest to the end of the DHI intervention delivery period to capture the most immediate and total effect of the engagement strategies. Intention-to-treat analyses were used in preference to complete-case analyses. For cluster RCTs, the study’s adjusted sample size was calculated by dividing it by the design effect. When no intracluster correlation coefficient was reported, it was assumed to be 0.05 [55]. Outcomes without sufficient data to calculate the SMD were excluded from the meta-analyses. RVE was performed on the SMDs (and associated variances) using the R package *robumeta* (R Foundation for Statistical Computing) [56]. A common within-study correlation (ρ) of 0.8 was assumed. To interpret ESs, we used guidance according to the Cohen *d* (small effect=0.2; moderate effect=0.5; large effect>0.8) [57].

#### Assessment of Heterogeneity

For the primary outcomes, heterogeneity was evaluated by examining forest plots for asymmetry. Statistical heterogeneity was also quantified by calculating the *I*^2^ statistic [51]. Where study heterogeneity was considerable (defined as *I*^2^>75%), we conducted subgroup analyses.

#### Subgroup and Sensitivity Analyses

Using multivariate meta-analyses, we performed subgroup analyses by use outcome measure (eg, time, log-ins, and activities completed), type of DHI (web-based program, activity tracker, mobile app, and telehealth), health risk (overweight and obesity, nutrition, and physical activity), and the study setting (closed, controlled data collection and delivery vs open, remote, and “real world” data collection and delivery). Sensitivity analyses were undertaken to determine whether the findings remained robust using different within-study correlations, whether studies were omitted that only reported medians rather than means (use meta-analysis only), and the effect of removing studies with an overall high ROB. Following a visual inspection of the meta-analysis forest plots, an additional sensitivity analysis removing outliers was undertaken (use meta-analysis only).

#### Meta-Regressions

To explore the association between individual strategies (BCTs and design features) and engagement outcomes, RVE meta-regressions were conducted where sufficient data were available. We deemed sufficient data to be available if there were ≥15 outcomes reported for an individual engagement strategy.

#### Secondary Outcome Synthesis

To synthesize the effects of the use of engagement strategies on the primary health risk outcome targeted by the DHI, we used the direction of the effect where available rather than statistical significance in accordance with SWiM (Synthesis Without Meta-Analysis) guidelines [58]. Similarly to previous reviews of nutrition, physical activity, and overweight and obesity interventions [52,59], we focused on a single measure of each health risk factor outcome from each study, which was selected based on a hierarchical criterion: validated measures over nonvalidated measures, global scores over individual constructs, measures that were deemed most relevant to answering the review question or were most commonly used among the included studies over other measures, and results from adjusted analyses over unadjusted analyses. Where a single measure could not be determined using the hierarchy, a web-based random number generator was used to select the measure to be included. For health risk measures that were reported at multiple time points, consistent with the outcomes chosen for engagement, the time point closest to the end of the intervention was extracted. For studies with multiple relevant intervention arms, we extracted findings from each intervention arm compared with the control arm.

## Results

### Search Results

**Figure 1:**
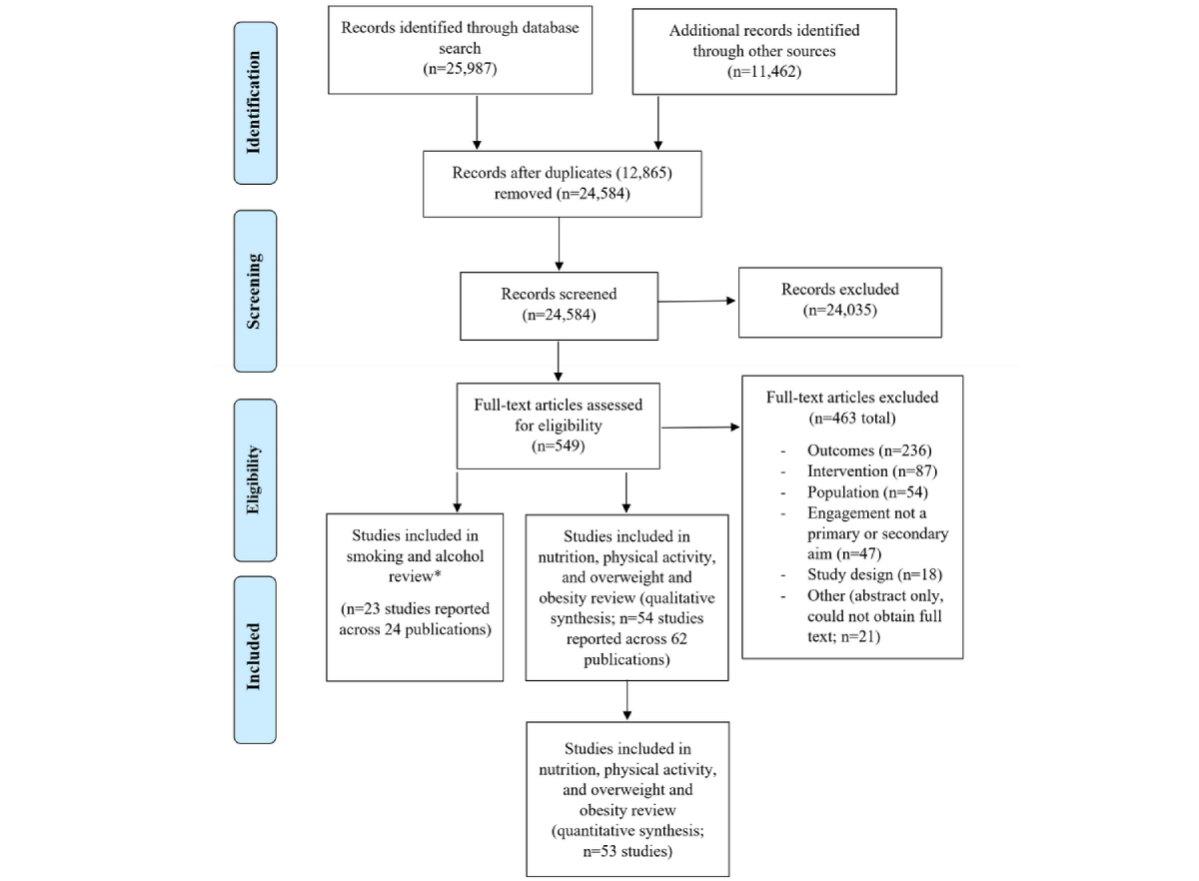
PRISMA (Preferred Reporting Items for Systematic Reviews and Meta-Analyses) flow diagram. *This review was conducted in conjunction with a mirror review of digital health interventions targeting smoking and alcohol consumption, the results of which will be reported elsewhere.

The searches resulted in 37,449 potentially relevant abstracts. Following the removal of duplicates and manual searching of reference lists of the included studies, 65.65% (24,584/37,449) of abstracts were retained for review, with 2.23% (549/24,584) undergoing full-text review (Figure 1).

### Included Studies and Participants

The characteristics of each study are described in Multimedia Appendix 3 [33,55,60-122]. There were 54 relevant studies reported across 62 publications included in the review. The studies were RCTs (46/54, 85%) [33,60-104], factorial RCTs (2/54, 4%) [105,106], or cluster RCTs (6/54, 11%) [55,107-111]. The sample sizes ranged from 10 to 8112 participants. The countries of origin included 50% (27/54) of studies from the United States [33,55,66,68,69,72-77,79-81,83,84,88,89,92-94,​^97^-^99^,^104^,^105^]; 28% (15/54) of studies from Australia [60,61,63-65,70,82,85,86,90,96,102,103,106,111]; 6% (3/54) of studies from the Netherlands [62,87,107]; 6% (3/54) of studies from the United Kingdom [67,71,110]; 6% (3/54) of studies from Canada [78,95,108]; and 2% (1/54) of studies each from China [109], Brazil [91], and Germany [101]. Overweight and obesity was the health risk primarily targeted in 57% (31/54) of the studies [33,55,62-65,67,69-72,74-76,79-81,83,​^84^,^87^,^89^,^91^-^94^,^98^-^100^,^104^,^107^,^112^], whereas 37% (20/54) of the studies primarily targeted physical activity [60,61,68,73,77,78,82,85,86,90,95,101-103,105,108-111,113] and 6% (3/54) primarily targeted nutrition [66,96,106]. The technologies used to deliver the DHIs included websites (33/54, 61%); mobile apps (11/54, 20%); SMS text messages (2/54, 4%); activity trackers (3/54, 6%); telehealth (1/54, 2%); a combination of an activity tracker, smart scale, and mobile app or website (1/54, 2%); a combination of an activity tracker, smart scale, and mobile app (1/54, 2%); a combination of a website and mobile app (1/54, 2%); and a combination of a website and telehealth (1/54, 2%). Most studies (45/54, 83%) had 2 arms included for the purpose of analysis, with 13% (7/54) of the studies including 3 arms [63,66,69,79,82,102,107] and 4% (2/54) of the studies using a 2 × 2 randomized factorial design [105,106]. A total of 65 intervention arms and 54 comparison arms were identified as relevant to the review.

### Strategies to Improve Engagement

#### Use of BCTs Identified as Engagement Strategies

For the purpose of the review, we considered any “unique” BCT (ie, a BCT identified in the intervention description of an included study arm but not in the comparison study arm) as a potential engagement strategy. A total of 61% (33/54) of the studies were identified as using one or more BCTs as an engagement strategy [33,55,61-69,71,72,76-80,83,88,89,92,93,​^98^,^99^,^102^-^109^,^111^]. The identified engagement strategy BCTs are listed in Multimedia Appendix 4 [33,55,62-64,​^67^,^69^,^71^,^72^,^74^-^76^,^79^-^81^,^83^,^84^,^87^,^89^,^91^-^94^,^97^-^100^,^104^,^107^,^116^,^118^]. In the remaining 39% (21/54) of the studies, no BCTs were identified as engagement strategies.

Examples of the types of BCTs identified as engagement strategies in the included studies can be found in Multimedia Appendix 2 [32,36]. The BCT *social support* was identified as an engagement strategy in 35% (19/54) of the studies [33,61-64,66,68,72,76,79,80,93,98,102-104,106,109,111], *reward and threat* (eg, providing material incentives) was identified in 22% (12/54) of the studies [55,67,78,92,93,99,102,105-107,109,111], *antecedents* (eg, changing the social environment to facilitate performance) was identified in 7% (4/54) of the studies [71,76,79,93], *associations* (eg, use of prompts) was identified in 13% (7/54) of the studies [64,68,77,88,93,102,108], *goals and planning* was identified in 11% (6/54) of the studies [33,64,68,93,102,114], *feedback and monitoring* was identified in 9% (5/54) of the studies [63,64,68,76,102], and *shaping knowledge* was identified in 7% (4/54) of the studies [76,79,93,109]. Other BCTs were identified in ≤6% (3/54) of the studies.

#### Use of Design Features Identified as Engagement Strategies

Similarly to BCTs, we considered any “unique” design feature (ie, a feature identified in the intervention description of an included study arm but not in the comparison study arm) as a potential engagement strategy. A total of 61% (33/54) of the studies were identified as using one or more design features as an engagement strategy [60-67,70,72,76,77,79,80,82,83,​^85^,^87^,^88^,^91^,^93^,^94^,^96^-^98^,^101^-^104^,^106^,^108^,^109^,^111^], with the remaining 39% (21/54) of the studies not using any design features as engagement strategies. The design features identified in each study are listed in Multimedia Appendix 4 [33,55,62-64,67,69,71,72,74-76,79-81,83,84,87,89,91-94,97-100,104,​^107^,^116^,^118^]. The design feature *enriched information environment* (eg, supplementary content and links, testimonials, videos, or games) was identified as an engagement strategy in 22% (12/54) of the studies [60,62,64,70,79,82,83,​^85^,^87^,^93^,^102^,^106^], *automated tailored feedback* was identified in 19% (10/54) of the studies [62-64,70,93,94,98,101,102,106], *automated follow-up messages* was identified in 11% (6/54) of the studies [62,64,65,76,77,88], *peer-to-peer access* was identified in 15% (8/54) of the studies [63,72,93,103,104,106,109,111], the use of *SMS text messages* was identified in 11% (6/54) of the studies [65,76,77,93,106,113], and the use of *telephone* calls was identified in 11% (6/54) of the studies [67,70,80,93,97,102]. Other design features were identified in ≤7% (4/54) of the studies.

#### Use of Other Strategies for Engagement

The strategies to improve engagement used in 19% (10/54) of the studies [73-75,81,84,86,90,95,100,110] were unable to be classified as BCTs or design features using the existing criteria. These types of strategies included comparing 2 different modes of delivering peer social support (web-based platform and in person) [74], providing the whole family with an activity tracker to support family group goal setting versus just 1 family member (child) [110], comparing different frequencies of prompts to enter tracking data into a web-based platform [84], comparing different framing of prompts (prevention- vs promotion-based prompts) [81], providing an activity tracker to record steps versus no activity tracker [86], providing digital scales and activity trackers to give to friends to enhance the social climate versus no additional equipment for friends [75], using smaller peer support groups to support group adhesion versus large generic discussion groups [73], comparing different frequencies of live and prerecorded physical activity sessions [95], and providing acceptance and commitment therapy versus no therapy [100].

#### Types of Engagement Outcomes

All but 2 of the studies (52/54, 96%) [94,96] reported use measures of engagement (ie, activities completed, log-ins, or time), and 54% (29/54) reported user experience measures of engagement [60-65,72,75,81-87,90,92,94-98,100-103,109,110]. Use outcomes of engagement were assessed objectively via data captured by the DHI (eg, analytics) or subjectively via self-report (eg, survey items). User experience outcomes were captured subjectively via self-report (eg, survey items) and objectively in one study using an eye-tracking device. In total, 403 engagement outcomes (n=219, 54.3% related to use and n=184, 45.7% related to user experience) were identified across the 54 included studies (Multimedia Appendix 4 [33,55,62-64,67,69,71,72,74-76,79-81,83,84,87,89,91-94,​^97^-^100^,^104^,^107^,^116^,^118^]).

#### Types of Health Risk Outcomes

Health risk outcomes were reported in 93% (50/54) of the studies. Of these 50 studies, 31 (62%) reported on overweight- and obesity-related outcomes, [33,55,62-65,67,69-72,​^74^-^77^,^79^-^81^,^83^,^85^-^87^,^89^,^91^-^95^,^98^,^99^,^104^], 29 (58%) reported on physical activity [55,61,64,65,68,70,73,77,78,82,​^85^-^88^,^90^-^93^,^95^,^97^,^100^-^105^,^107^-^109^,^111^], and 14 (28%) reported on nutrition outcomes [64,65,70,71,77,​^83^,^87^,^91^,^93^,^96^,^97^,^104^,^106^,^107^]. Outcomes related to overweight and obesity included BMI, BMI z-score, weight change, waist circumference, percentage of weight loss, and the proportion of participants with clinically significant weight loss. Outcomes related to physical activity included steps (number, mean/day, and change in daily step counts), moderate to vigorous physical activity (min, mean min/day, and mean 10-min bouts/wk), physical activity (total and min/wk), physical activity level score, kilocalories expended per kilogram, and the Godin Leisure-Time Exercise Questionnaire. Outcomes related to nutrition included fruit, vegetable, sweet snack, and energy (calorie and kJ/day) intake; percentage of calories from saturated fat; and diet quality.

### Methodological Quality of Studies: ROB of Included Studies

**Figure 2:**
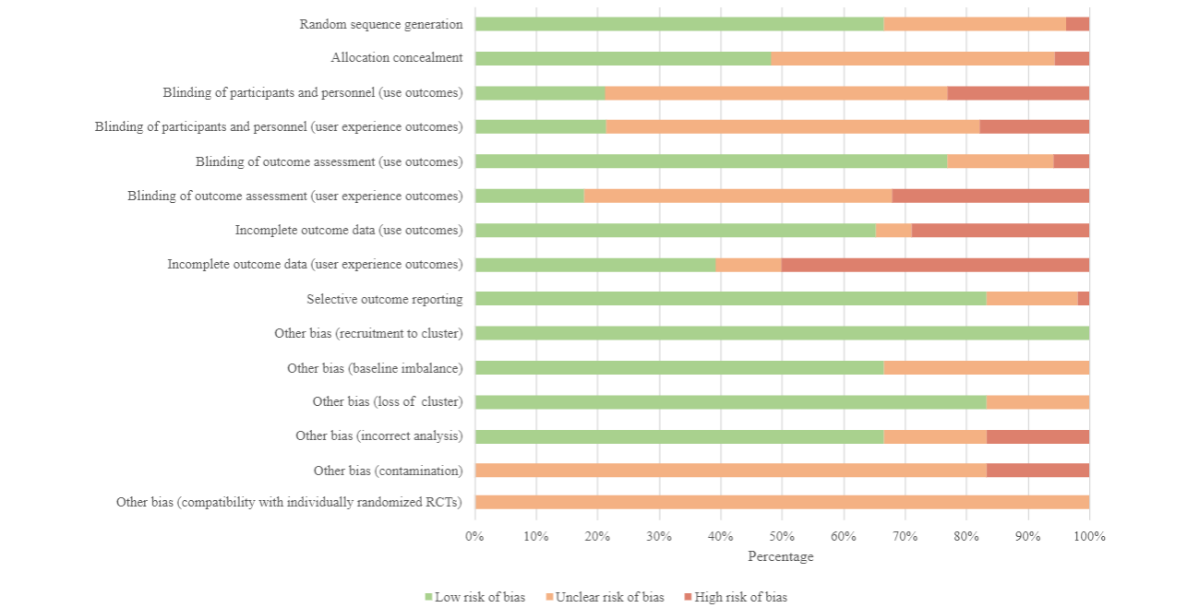
Risk-of-bias summary. RCT: randomized controlled trial.

For the included engagement outcomes (use and user experience), the ROB is reported separately in Multimedia Appendix 5 [33,55,60-64,66-111,114,116-118,122]. Figure 2 shows the percentage of studies with high, unclear, and low ROB for each domain. An assessment of overall bias found most studies (30/54, 56%) to be at low risk, 39% (21/54) of the studies to be unclear [33,55,65,66,68,72,74,76,81,82,84,86,​^89^,^92^,^95^,^97^-^100^,^102^,^104^], and 6% (3/54) of the studies to be at high risk [85,87,101].

Most studies were deemed low risk for “random sequence generation” (36/54, 67%) [55,60-62,64,65,67,69-73,​^75^,^77^-^82^,^85^-^88^,^90^,^91^,^93^,^94^,^96^,^97^,^100^,^102^,^103^,^106^,^108^,^110^,^111^] and “selective outcome reporting” (45/54, 83%) [33,55,60-76,78-85,87,90-97,101-103,105-107,109-111]. For use outcomes, most studies were deemed low risk for “incomplete outcome data” (34/52, 65%) [33,60,61,63,66-70,73,75-80,83,84,88-93,98-100,103,105-107,109-111] and “blinding of outcome assessment” (40/52, 77%) [33,55,60-67,69-73,75-77,79,80,82,83,87,90-93,95,98-100,103-111]. For user experience outcomes, the ROB for “incomplete outcome data” was mixed, with half (14/28, 50%) of the studies rated as high risk [62,64,65,72,81,82,85-87,95,96,98,101,102]. For user experience outcomes, the ROB for “blinding of outcome assessment” was also mixed, with 50% (14/28) of the studies rated as unclear [61,65,81,84,86,90,92,94-98,100,103]. Almost half (26/54, 48%) of all the studies had low risk for “allocation concealment” [60,65,67,70,71,75,77-79,82,83,​^85^-^88^,^90^,^91^,^93^,^96^,^100^,^102^,^106^,^108^-^111^], and just over half (29/52, 56%) were unclear for “blinding of participants and personnel” use outcomes [33,55,60,65,66,72-74,76-78,​^81^,^82^,^84^,^86^,^88^-^90^,^92^,^95^,^97^-^100^,^102^-^104^,^110^,^111^], whereas 61% (17/28) of the studies were unclear for “blinding of participants and personnel” user experience outcomes [60,65,72,81,82,84,86,90,92,94-98,100,102,103].

Of the 6 cluster RCTs [55,107-111], most were rated as low for “baseline imbalance” (n=4, 67%) [55,107,109,111], “recruitment to cluster” (n=6, 100%), “loss of cluster” (n=5, 83%) [107-111], and “incorrect analysis” (n=4, 67%) [55,107,109,111]. For “other bias (contamination),” most studies (5/6, 83%) were rated as unclear [55,107-109,111]. For “compatibility with individually randomized RCTs,” all cluster trials (6/6, 100%) were rated as having an unclear ROB.

### Primary Outcomes

#### Overall Effectiveness of Strategies to Increase Use Outcomes of Engagement

##### Overview

For the meta-analysis of use outcomes, 201 outcomes from 94% (51/54) of the studies with 17,828 participants were included. The excluded use outcomes and studies and reasons for exclusion are summarized in Multimedia Appendix 6 [33,55,61,66-80,84,85,88,89,91,93,94,96,98,99,102-108,111,118].

Details of the study arms, SMDs for each comparison condition, and 95% CIs are provided in Multimedia Appendix 4 [33,55,62-64,67,69,71,72,74-76,79-81,83,84,87,89,91-94,97-100,104,107,116,118]. Pooled analysis of the 51 included studies found a very low-certainty level of evidence of a small-to-moderate positive effect of the use of any strategy (including BCTs and design features) on DHI use (SMD=0.33, 95% CI 0.20-0.46; *I*^2^=85.9%; *P*<.001; Table S1 in Multimedia Appendix 7). The certainty of evidence (using GRADE) was assessed as very-low because of downgrading by 1 level for inconsistency (substantial heterogeneity; *I*^2^=85.9%), 1 level for indirectness, and 1 level for imprecision (Table S1 in Multimedia Appendix 7).

##### Subgroup and Sensitivity Analysis

In total, 2 sensitivity tests were undertaken. One was to determine whether the findings remained robust when using different within-study correlations, and the second was to determine whether the findings remained robust when omitting studies that only reported medians rather than means. The results are displayed in Tables S2 and S3 in Multimedia Appendix 7. For both sensitivity tests, there were only minor differences between the 2 sets of results. Inspection of the forest plot (Figure S1 in Multimedia Appendix 7) indicated that an outlier, the study by Alley et al [60], could have been driving the significant results as the SMD for this study was much larger than that for any other study. However, the *P* value remained significant after a post hoc sensitivity analysis excluding the study by Alley et al [60] from the meta-analysis (SMD=0.30, 95% CI 0.18-0.43; *P*<.001; Table S4 in Multimedia Appendix 7). The results of an additional sensitivity analysis excluding studies with an overall high ROB found that the point estimate increased slightly (SMD=0.36, 95% CI 0.23-0.49; *P*<.001; Table S5 in Multimedia Appendix 7). The subgroup analyses found no substantial differences in the SMD according to health risk outcome targeted, DHI type, type of use outcome, or study setting (Tables S6-S9 in Multimedia Appendix 7).

#### Associations Between Individual Strategies and Use Outcomes of Engagement

##### Use of Individual BCTs as Strategies to Improve Engagement

For those studies reporting on use outcomes, 112 outcomes from 63% (32/51) of the studies that used one or more BCTs as an engagement strategy were included [33,55,61-64,66-69,71,72,76-80,83,88,89,92,93,98,99,102-109,111]. There were no BCTs coded as engagement strategies for the remaining 89 outcomes from 41% (21/51) of the studies. To determine whether there was an association between the use of individual BCTs and use outcomes of engagement, we undertook 9 meta-regressions based on there being a sufficient number of outcomes reported for a particular engagement strategy. Sufficient outcomes were available for the engagement strategy *social support* (63 outcomes from 19/51, 37% of the studies), followed by *reward and threat* (49 outcomes from 11/51, 22% of the studies), *goals and planning* (26 outcomes from 6/51, 12% of the studies), *associations* (24 outcomes from 7/54, 13% of the studies), *feedback and monitoring* (21 outcomes from 5/51, 10% of the studies), *scheduled consequences* (20 outcomes from 2/51, 4% of the studies), *antecedents* (19 outcomes from 4/51, 8% of the studies), *repetition and substitution* (17 outcomes from 3/51, 6% of the studies), and *shaping knowledge* (15 outcomes from 4/51, 8% of the studies). The results of the meta-regressions can be found in Table S10 in Multimedia Appendix 7. Small-to-moderate, statistically significant associations were detected for the use of the BCTs *social support* (ES=0.40, 95% CI 0.14-0.66; *I*^2^=85.2%; *P*<.001) and *shaping knowledge* (ES=0.39, 95% CI 0.03-0.74; *I*^2^=85.4%; *P*=.03) as engagement strategies to improve DHI use but not for any other BCTs.

##### Use of Individual Design Features as Strategies to Improve Engagement

We identified 111 outcomes from 61% (31/51) of the studies that used one or more design features as an engagement strategy, and they were included in the meta-regressions [60-67,70,72,76,77,79,80,82,83,85,87,88,91,93,97,98,101-104,106,108,109,111]. There were no design features coded as engagement strategies for the remaining 90 outcomes from 43% (22/51) of the studies. Several meta-regressions were undertaken to explore any associations between specific design features and use engagement outcomes. There was a sufficient number of outcomes available for the design features *enriched information environment* (57 outcomes from 12/51, 24% of the studies), *automated tailored feedback* (34 outcomes from 9/51, 18% of the studies), *telephone contact* (25 outcomes from 6/51, 12% of the studies), *email* (20 outcomes from 5/51, 10% of the studies), *peer-to-peer access* (20 outcomes from 8/51, 16% of the studies), and *automated follow-up messages* (17 outcomes from 6/51, 12% of the studies). The results of the meta-regressions can be found in Table S10 in Multimedia Appendix 7. No statistically significant effects were detected for any of the meta-regressions.

#### Overall Effectiveness of Strategies to Increase User Experience Outcomes of Engagement

##### Overview

For the meta-analysis of engagement outcomes of user experience, 178 outcomes from 44% (24/54) of the studies were included. The excluded outcomes and studies and reasons for exclusion are summarized in Multimedia Appendix 6 [33,55,61,66-80,84,85,88,89,91,93,94,96,98,99,102-108,111,118].

Details of the study arms, SMDs for each comparison condition, and 95% CIs are provided in Multimedia Appendix 4 [33,55,62-64,67,69,71,72,74-76,79-81,83,84,87,89,91-94,97-100,104,107,116,118]. The results of the meta-analysis revealed that there was very low-certainty of evidence for a small-to-moderate positive effect of strategies on user experience outcomes of engagement (SMD=0.29, 95% CI 0.07-0.52; *I*^2^=78%; *P*=.01; Table S11 in Multimedia Appendix 7). The certainty of evidence was assessed as very low because of downgrading by 1 level for inconsistency (substantial heterogeneity; *I*^2^=78%), 1 level for indirectness, and 1 level for imprecision (Table S11 in Multimedia Appendix 7).

##### Subgroup and Sensitivity Analysis

Sensitivity tests to determine whether the findings remained robust using different within-study correlations are provided in Table S12 in Multimedia Appendix 7. Little to no variation was detected. The results of a sensitivity analysis excluding studies with an overall high ROB found that the overall point estimate increased (SMD=0.34, 95% CI 0.08-0.60; *P*=.01; Table S13 in Multimedia Appendix 7). The subgroup analyses exploring differences by DHI type found significantly lower SMDs for studies using mobile apps (SMD=−0.46, 95% CI −0.83 to −0.10; *P*=.02) and SMS text messages (SMD=−0.43, 95% CI −0.73 to −0.14; *P*=01; Table S16 in Multimedia Appendix 7). The subgroup analyses exploring differences by health risk outcome targeted and study setting found no significant differences in the SMDs (Tables S15-S17 in Multimedia Appendix 7).

#### Associations Between Individual Strategies and User Experience Outcomes of Engagement

##### Use of Individual BCTs as Strategies to Improve Engagement

For those studies reporting user experience outcomes, we identified 60 outcomes from 33% (8/24) of the studies using one or more BCTs as an engagement strategy [62-64,72,83,92,102,109]. There were no BCTs coded as engagement strategies for the remaining 117 outcomes from 67% (16/24) of the studies. To determine whether there was an association between the use of BCTs and user experience outcomes, we undertook 8 meta-regressions for the BCTs *social support* (50 outcomes from 6/24, 25% of the studies), *feedback and monitoring* (42 outcomes from 3/24, 13% of the studies), *self-belief* (39 outcomes from 2/24, 8% of the studies), *reward and threat* (36 outcomes from 3/24, 13% of the studies), *goals and planning* (36 outcomes from 2/24, 8% of the studies), *associations* (36 outcomes from 2/24, 8% of the studies), *repetition and substitution* (30 outcomes from 1/24, 4% of the studies), and *natural consequences* (30 outcomes from 1/24, 4% of the studies). The results of the meta-regression can be found in Table S14 in Multimedia Appendix 7. A moderate-to-large statistically significant association was detected for the use of the BCT *social support* (ES=0.70, 95% CI 0.18-1.22; *I*^2^=68.3%; *P*=.01), with small-to-moderate associations found for *repetition and substitution* (ES=0.29, 95% CI 0.05-0.53; *I*^2^=78.3%; *P*=.02) and *natural consequences* (ES=0.29, 95% CI 0.05-0.53; *I*^2^=78.3%; *P*=.02) as engagement strategies to improve DHI user experience but not for any other BCTs.

##### Use of Individual Design Features as Strategies to Improve Engagement

For those studies reporting on user experience outcomes, we identified 113 outcomes from 67% (16/24) of the studies using one or more design features as an engagement strategy [60,62-65,72,82,83,85,87,94,96,97,101,102,109]. There were no design features coded as engagement strategies in 64 outcomes from 33% (8/24) of the studies. We undertook 6 meta-regressions exploring associations between individual design features and user experience outcomes. These included the *automated functions of enriched information environment* (68 outcomes from 8/24, 33% of the studies), *automated tailored feedback* (49 outcomes from 6/24, 25% of the studies), *telephone contact* (31 outcomes from 2/24, 8% of the studies), *email* (30 outcomes from 1/24, 4% of the studies), *automated follow-up messages* (27 outcomes from 3/24, 13% of the studies) [62,64,65], and the *supplementary design feature SMS text messages* (20 outcomes from 1/24, 4% of the studies) [65]. The results of the meta-regression can be found in Table S14 in Multimedia Appendix 7. Small-to-moderate statistically significant associations were detected for the use of *email* (ES=0.29, 95% CI 0.05-0.53; *I*^2^=78.3%; *P*=.02) and *SMS text messages* (ES=0.34, 95% CI 0.11-0.57; *I*^2^=78.8%; *P*=.01) as engagement strategies to improve DHI user experience but not for any other design features.

### Secondary Outcomes

The impact of strategies on health risk outcomes for each study is reported in Multimedia Appendix 4 [33,55,62-64,67,69,71,72,74-76,79-81,83,84,87,89,91-94,97-100,104,107,116,118]. Of the included studies (N=54), 7% (4/54) did not report any health outcomes at follow-up [60,66,84,110], and health outcomes could not be extracted for 2% (1/54) of the studies as they did not report these outcomes separately for the intervention and control arms [107]. In total, 13% (7/54) of the studies contributed findings from more than one intervention arm [63,69,79,82,102,105,106]. Overall, among the 57% (31/54) of studies reporting overweight- and obesity-related outcomes, 41% (14/34) of the outcomes were positive and in the hypothesized direction (ie, improvements in the intervention arm compared with the control arm). Among the 54% (29/54) of studies reporting physical activity outcomes, 73% (24/33) of the outcomes were positive and in the hypothesized direction. Among the 26% (14/54) of studies reporting nutrition-related outcomes, 47% (7/15) of the outcomes were positive and in the hypothesized direction.

## Discussion

### Summary of Findings

The primary purpose of this review was to assess the effectiveness of strategies to improve engagement with DHIs targeting the health risk factors of chronic diseases (overweight and obesity, physical activity, and nutrition) and explore the association between individual strategies (BCTs and design features) and engagement. Overall, our review found evidence that the use of strategies (such as additional BCTs or design features) improves engagement with DHIs for both use and user experience outcomes; however, the quality of evidence rating was very low. When exploring associations between individual strategies and engagement, the use of the BCTs *social support* and *shaping knowledge* was found to be significantly associated with improvements in use measures of engagement, whereas *social support, repetition and substitution*, and *natural consequences* were significantly associated with improvements in user experience measures of engagement. The design features *email* and *SMS text messages* were also found to be significantly associated with user experience outcomes of engagement.

### Completeness and Applicability of Evidence

This review included 54 trials with between 10 and 8112 participants. Most of the studies were conducted in the United States (27/54, 50%) and Australia (15/54, 28%); therefore, the generalization of the results to other countries, particularly low- and middle-income countries, may be limited. Most studies primarily targeted overweight or obesity (31/54, 57%) followed by physical activity (20/54, 37%), with only 6% (3/54) of the trials targeting nutrition. Although all of the overweight and obesity studies (n=31) included a nutritional component, the findings of this review may not be as applicable to DHIs focusing solely on nutrition. The most common technology used to deliver DHIs was websites (33/54, 61%); therefore, our results may not be as applicable to other types of DHIs (eg, mobile health interventions).

### Comparisons With Other Reviews

As this systematic review was the first to use multivariate meta-analysis to assess the overall effectiveness of strategies to increase engagement with DHIs targeting overweight and obesity, nutrition, and physical activity, our ability to directly compare our findings with those of other reviews is limited. We could only identify 1 previous systematic review using a meta-analysis to determine the effect of strategies on engagement with DHIs. This review by Alkhaldi et al [31] included DHIs targeting physical or mental health and measured only the effects of digital strategies (primarily the use of *email* or *telephone*) to prompt DHI use on use-based engagement measures. Similarly to our findings, this review found a small overall effect (relative risk=1.27, 95% CI 1.01-1.60; *I*^2^=71%) [31].

Our finding that the use of the BCT *social support* can improve use and user experience engagement with DHIs is consistent with those of several previous systematic reviews [37,123,124]. However, the findings that *shaping knowledge*, *repetition and substitution*, and *natural consequences* are associated with engagement appear to be more novel. Only 1 previous review by Perski et al [20] has categorized engagement strategies as BCTs. This review used narrative critical interpretative synthesis and, in addition to identifying the BCT *social support*, found the BCTs *goals and planning*, *feedback and monitoring*, and *rewards and incentives* to be positively associated with engagement. The contrast between the review by Perski et al [20] and our review is likely due to differences in methodology (ie, narrative synthesis vs meta-regressions) and the vastly greater body of literature now available since the conduct of the review by Perski et al [20] in 2015.

Our review findings indicate that design features, including email and SMS text messages, may be promising strategies for improving user experience engagement with DHIs. Although the meta-regressions indicated that these strategies provided small positive effects in increasing use outcomes of engagement, these were not statistically significant. The use of email as an engagement strategy was also reported in a previous narrative systematic review by Brouwer et al [37]; however, this was associated with improvements in use-based outcomes (promoting repeated visits to DHIs). To our knowledge, there are no other systematic reviews that report the use of SMS text messages as a strategy to improve engagement with DHIs; hence, this represents a novel finding. Although email and SMS text messages are often used to prompt users to log in to DHIs or complete activities [31], such design features may also be used to deliver an array of content (including professional or social support), encourage the continuation of behavior change, or set goals [20], all of which may lend itself to increases in engagement via different mechanisms. Therefore, this finding highlights the differential impact of design features on use and user experience engagement outcomes, which could be explored further in future reviews.

Finally, the impact of strategies on health risk outcomes was mixed, with only 41% (14/34) of overweight- and obesity-related outcomes, 73% (24/33) of physical activity–related outcomes, and 47% (7/15) of nutrition-related outcomes found to be positive and in the hypothesized direction. Although previous reviews have demonstrated the effectiveness of DHIs in improving health outcomes [25-27], this is the first review to synthesize the effects of strategies to improve engagement with DHIs on health outcomes, and so comparisons cannot be drawn.

### Quality of Evidence

The certainty of evidence (determined using GRADE) in this review was assessed as very low. The evidence was downgraded based on high levels of unexplained heterogeneity (inconsistency), engagement being an exploratory or secondary outcome in the included studies (indirectness), and the presence of wide 95% CIs (imprecision).

When assessing the study ROB, just 6% (3/54) of the studies were deemed to be high risk overall. Regarding individual domains of risk, incomplete outcome data (user experience outcomes) was deemed to be high risk in 50% (14/28) of the studies. This was commonly due to the high study attrition rates. As ≥50% of the studies were assessed as unclear for blinding of participants and personnel (usage; 29/52, 56%), for blinding of participants and personnel (user experiences; 17/28, 61%), blinding of outcome assessment (user experience; 14/28, 50%), other bias (contamination; 5/6, 83%), and compatibility with randomized trials (for cluster RCTs; 6/6, 100%), improved reporting for these ROB domains in future trials is recommended.

### Strengths, Limitations, and Implications

The findings of this review should be interpreted with respect to its strengths and limitations. Strengths include the use of the recommended methodology for Cochrane systematic reviews; the use of a comprehensive search strategy, which included screening >24,000 records; and the restriction to studies using an RCT design, ensuring the synthesis of the highest quality of evidence available for assessing causality. In addition, unlike many systematic reviews of DHI engagement, our review addressed both use and user experience engagement outcomes, used robust meta-analyses, and used previously defined coding systems to classify engagement strategies. Overall, we identified >250 outcome measures for engagement, emphasizing the need for researchers to continue to move toward a more refined, standardized approach to measuring engagement using validated tools where possible. This may assist in reducing the heterogeneity between trials and improving the quality of evidence available for future systematic reviews.

A limitation of this review is that we isolated individual BCTs and design features that were unique to the intervention arms in the included studies. However, we did not have the ability to separate any potential interactive effects of combinations of BCTs and design features. An analytic methodology to attempt to identify any synergistic effects of BCTs and design features on health outcomes was developed by van Genugten et al [125], which could be an option for future reviews aiming to identify how multiple strategies for improving engagement may influence outcomes of interest. We also did not explore the dose of strategies delivered to improve engagement, which is likely an important factor when considering the effectiveness of engagement strategies [20]. This review sought to identify associations between engagement strategies (coded via overarching BCTs) and engagement outcomes. Further research exploring the impact of discrete techniques within BCTs on engagement outcomes may be warranted to optimize the effect of such strategies on use and user experience outcomes. Finally, a number of engagement outcomes reported in the included studies were excluded from the analyses. This was often due to insufficient data being reported (eg, no measure of variability or numbers per arm not reported). As such, it is recommended that future trials using DHIs report high-quality engagement data to further advance the evidence regarding the overall and individual effectiveness of strategies to increase engagement [126].

Finally, the ability to interpret the real-world significance of reported improvements in engagement outcomes remains somewhat limited as the optimal amount of engagement required is likely to differ based on the design and intent of individual DHIs and also because of the premise that the quality and nature of engagement may be more important than quantity (ie, more may not always be better) [28]. It has been proposed that an optimal level of engagement is the amount that is required to achieve the desired effects of the DHI [28]. Consistent with previous recommendations [18,20,28], to better determine the significance of improvements in engagement outcomes in future systematic reviews, trials testing the use of engagement strategies should aim to specify the level of improvement in engagement that they aim to achieve a priori (eg, based on the level of engagement demonstrated to achieve health outcomes in previous effectiveness trials of the DHI) and report whether this was achieved alongside validated use and user experience outcomes of engagement.

### Conclusions

Overall, the use of engagement strategies may improve both use and user experience engagement with DHIs targeting overweight and obesity, physical activity, and nutrition; however, the true effect is unknown because the quality of evidence was very low. As individual strategies, the use of the BCTs *social support*, s*haping knowledge*, *repetition and substitution*, and *natural consequences* and the design features *email* and *SMS text messages* was found to be associated with improved measures of engagement. Such findings may be useful to policymakers and practitioners tasked with selecting, designing, or implementing DHIs to support population health. Given the extensive range of possible strategies that can be used to improve engagement with DHIs, this review provides support for investment in further exploration of the role of these BCTs and design features in engaging participants in the use of DHIs targeting such health outcomes. Future trials should use standardized and validated measures of engagement where available, report on both use and user experience measures of engagement, and prespecify minimum targets for engagement to better identify specific strategies for optimizing engagement with such DHIs.

## Appendix

Multimedia Appendix 1 Search terms.

Multimedia Appendix 2 Engagement strategy codes and examples.

Multimedia Appendix 3 Characteristics of the included studies.

Multimedia Appendix 4 Study arms and outcome effect sizes.

Multimedia Appendix 5 Risk-of-bias summary.

Multimedia Appendix 6 Excluded studies and outcomes.

Multimedia Appendix 7 Meta-analysis and meta-regression results.

Multimedia Appendix 8 PRISMA Checklist.

## Abbreviations

BCT: behavior change technique
DHI: digital health intervention
ES: effect size
GRADE: Grading of Recommendations Assessment, Development, and Evaluation
PRISMA: Preferred Reporting Items for Systematic Reviews and Meta-Analyses
RCT: randomized controlled trial
ROB: risk of bias
RVE: robust variance estimation
SMD: standardized mean difference
SWiM: Synthesis Without Meta-Analysis
WHO: World Health Organization

## References

[1] GBD 2017 Causes of Death Collaborators (2018). Global, regional, and national age-sex-specific mortality for 282 causes of death in 195 countries and territories, 1980-2017: a systematic analysis for the Global Burden of Disease Study 2017. Lancet.

[2] GBD 2016 DALYs and HALE Collaborators (2017). Global, regional, and national disability-adjusted life-years (DALYs) for 333 diseases and injuries and healthy life expectancy (HALE) for 195 countries and territories, 1990-2016: a systematic analysis for the Global Burden of Disease Study 2016. Lancet.

[3] Lim SS, Vos T, Flaxman AD, Danaei G, Shibuya K, Adair-Rohani H, Amann M, Anderson HR, Andrews KG, Aryee M, Atkinson C, Bacchus LJ, Bahalim AN, Balakrishnan K, Balmes J, Barker-Collo S, Baxter A, Bell ML, Blore JD, Blyth F, Bonner C, Borges G, Bourne R, Boussinesq M, Brauer M, Brooks P, Bruce NG, Brunekreef B, Bryan-Hancock C, Bucello C, Buchbinder R, Bull F, Burnett RT, Byers TE, Calabria B, Carapetis J, Carnahan E, Chafe Z, Charlson F, Chen H, Chen JS, Cheng AT, Child JC, Cohen A, Colson KE, Cowie BC, Darby S, Darling S, Davis A, Degenhardt L, Dentener F, Des JD, Devries K, Dherani M, Ding EL, Dorsey ER, Driscoll T, Edmond K, Ali SE, Engell RE, Erwin PJ, Fahimi S, Falder G, Farzadfar F, Ferrari A, Finucane MM, Flaxman S, Fowkes FG, Freedman G, Freeman MK, Gakidou E, Ghosh S, Giovannucci E, Gmel G, Graham K, Grainger R, Grant B, Gunnell D, Gutierrez HR, Hall W, Hoek HW, Hogan A, Hosgood HD, Hoy D, Hu H, Hubbell BJ, Hutchings SJ, Ibeanusi SE, Jacklyn GL, Jasrasaria R, Jonas JB, Kan H, Kanis JA, Kassebaum N, Kawakami N, Khang Y, Khatibzadeh S, Khoo J, Kok C, Laden F, Lalloo R, Lan Q, Lathlean T, Leasher JL, Leigh J, Li Y, Lin JK, Lipshultz SE, London S, Lozano R, Lu Y, Mak J, Malekzadeh R, Mallinger L, Marcenes W, March L, Marks R, Martin R, McGale P, McGrath J, Mehta S, Mensah GA, Merriman TR, Micha R, Michaud C, Mishra V, Mohd HK, Mokdad AA, Morawska L, Mozaffarian D, Murphy T, Naghavi M, Neal B, Nelson PK, Nolla JM, Norman R, Olives C, Omer SB, Orchard J, Osborne R, Ostro B, Page A, Pandey KD, Parry CD, Passmore E, Patra J, Pearce N, Pelizzari PM, Petzold M, Phillips MR, Pope D, Pope CA, Powles J, Rao M, Razavi H, Rehfuess EA, Rehm JT, Ritz B, Rivara FP, Roberts T, Robinson C, Rodriguez-Portales JA, Romieu I, Room R, Rosenfeld LC, Roy A, Rushton L, Salomon JA, Sampson U, Sanchez-Riera L, Sanman E, Sapkota A, Seedat S, Shi P, Shield K, Shivakoti R, Singh GM, Sleet DA, Smith E, Smith KR, Stapelberg NJC, Steenland K, Stöckl H, Stovner LJ, Straif K, Straney L, Thurston GD, Tran JH, Van DR, van DA, Veerman JL, Vijayakumar L, Weintraub R, Weissman MM, White RA, Whiteford H, Wiersma ST, Wilkinson JD, Williams HC, Williams W, Wilson N, Woolf AD, Yip P, Zielinski JM, Lopez AD, Murray CJL, Ezzati M, AlMazroa MA, Memish ZA (2012). A comparative risk assessment of burden of disease and injury attributable to 67 risk factors and risk factor clusters in 21 regions, 1990-2010: a systematic analysis for the Global Burden of Disease Study 2010. Lancet.

[4] Schoeppe S, Alley S, Van Lippevelde W, Bray NA, Williams SL, Duncan MJ, Vandelanotte C (2016). Efficacy of interventions that use apps to improve diet, physical activity and sedentary behaviour: a systematic review. Int J Behav Nutr Phys Act.

[5] Widmer RJ, Collins NM, Collins CS, West CP, Lerman LO, Lerman A (2015). Digital health interventions for the prevention of cardiovascular disease: a systematic review and meta-analysis. Mayo Clin Proc.

[6] Joiner KL, Nam S, Whittemore R (2017). Lifestyle interventions based on the diabetes prevention program delivered via eHealth: A systematic review and meta-analysis. Prev Med.

[7] (2019). WHO guideline: recommendations on digital interventions for health system strengthening. World Health Organization.

[8] (2016). Monitoring and evaluating digital health interventions: a practical guide to conducting research and assessment. World Health Organization.

[9] Bastawrous A, Armstrong MJ (2013). Mobile health use in low- and high-income countries: an overview of the peer-reviewed literature. J R Soc Med.

[10] Payne HE, Lister C, West JH, Bernhardt JM (2015). Behavioral functionality of mobile apps in health interventions: a systematic review of the literature. JMIR Mhealth Uhealth.

[11] Zarnowiecki D, Mauch CE, Middleton G, Matwiejczyk L, Watson WL, Dibbs J, Dessaix A, Golley RK (2020). A systematic evaluation of digital nutrition promotion websites and apps for supporting parents to influence children's nutrition. Int J Behav Nutr Phys Act.

[12] Kohl LF, Crutzen R, de Vries NK (2013). Online prevention aimed at lifestyle behaviors: a systematic review of reviews. J Med Internet Res.

[13] Jahangiry L, Farhangi MA, Shab-Bidar S, Rezaei F, Pashaei T (2017). Web-based physical activity interventions: a systematic review and meta-analysis of randomized controlled trials. Public Health.

[14] Beleigoli AM, Andrade AQ, Cançado AG, Paulo MN, Diniz MD, Ribeiro AL (2019). Web-based digital health interventions for weight loss and lifestyle habit changes in overweight and obese adults: systematic review and meta-analysis. J Med Internet Res.

[15] Berry R, Kassavou A, Sutton S (2021). Does self-monitoring diet and physical activity behaviors using digital technology support adults with obesity or overweight to lose weight? A systematic literature review with meta-analysis. Obes Rev.

[16] Shi Y, Wakaba K, Kiyohara K, Hayashi F, Tsushita K, Nakata Y (2022). Effectiveness and components of web-based interventions on weight changes in adults who were overweight and obese: a systematic review with meta-analyses. Nutrients.

[17] Grady A, Wolfenden L, Rissel C, Green S, Reilly K, Yoong SL (2019). Effectiveness of a dissemination strategy on the uptake of an online menu planning program: a controlled trial. Health Promot J Austr.

[18] Michie S, Yardley L, West R, Patrick K, Greaves F (2017). Developing and evaluating digital interventions to promote behavior change in health and health care: recommendations resulting from an international workshop. J Med Internet Res.

[19] Morrison LG (2015). Theory-based strategies for enhancing the impact and usage of digital health behaviour change interventions: a review. Digit Health.

[20] Perski O, Blandford A, West R, Michie S (2017). Conceptualising engagement with digital behaviour change interventions: a systematic review using principles from critical interpretive synthesis. Transl Behav Med.

[21] Short CE, DeSmet A, Woods C, Williams SL, Maher C, Middelweerd A, Müller AM, Wark PA, Vandelanotte C, Poppe L, Hingle MD, Crutzen R (2018). Measuring engagement in eHealth and mHealth behavior change interventions: viewpoint of methodologies. J Med Internet Res.

[22] Short CE, Rebar AL, Plotnikoff RC, Vandelanotte C (2015). Designing engaging online behaviour change interventions: a proposed model of user engagement. Eur J Health Psychol.

[23] Kelders SM, Kok RN, Ossebaard HC, Van Gemert-Pijnen JE (2012). Persuasive system design does matter: a systematic review of adherence to web-based interventions. J Med Internet Res.

[24] Retention rate on day 30 of mobile app installs worldwide in 3rd quarter 2022, by category. Statistica.

[25] Donkin L, Christensen H, Naismith SL, Neal B, Hickie IB, Glozier N (2011). A systematic review of the impact of adherence on the effectiveness of e-therapies. J Med Internet Res.

[26] Mclaughlin M, Delaney T, Hall A, Byaruhanga J, Mackie P, Grady A, Reilly K, Campbell E, Sutherland R, Wiggers J, Wolfenden L (2021). Associations between digital health intervention engagement, physical activity, and sedentary behavior: systematic review and meta-analysis. J Med Internet Res.

[27] Delaney T, Mclaughlin M, Hall A, Yoong SL, Brown A, O'Brien K, Dray J, Barnes C, Hollis J, Wyse R, Wiggers J, Sutherland R, Wolfenden L (2021). Associations between digital health intervention engagement and dietary intake: a systematic review. Nutrients.

[28] Yardley L, Spring BJ, Riper H, Morrison LG, Crane DH, Curtis K, Merchant GC, Naughton F, Blandford A (2016). Understanding and promoting effective engagement with digital behavior change interventions. Am J Prev Med.

[29] Machado GC, Pinheiro MB, Lee H, Ahmed OH, Hendrick P, Williams C, Kamper SJ (2016). Smartphone apps for the self-management of low back pain: a systematic review. Best Pract Res Clin Rheumatol.

[30] Hutchesson MJ, Gough C, Müller AM, Short CE, Whatnall MC, Ahmed M, Pearson N, Yin Z, Ashton LM, Maher C, Staiano AE, Mauch CE, DeSmet A, Vandelanotte C (2021). eHealth interventions targeting nutrition, physical activity, sedentary behavior, or obesity in adults: a scoping review of systematic reviews. Obes Rev.

[31] Alkhaldi G, Hamilton FL, Lau R, Webster R, Michie S, Murray E (2016). The effectiveness of prompts to promote engagement with digital interventions: a systematic review. J Med Internet Res.

[32] Michie S, Ashford S, Sniehotta FF, Dombrowski SU, Bishop A, French DP (2011). A refined taxonomy of behaviour change techniques to help people change their physical activity and healthy eating behaviours: the CALO-RE taxonomy. Psychol Health.

[33] Webber KH, Gabriele JM, Tate DF, Dignan MB (2010). The effect of a motivational intervention on weight loss is moderated by level of baseline controlled motivation. Int J Behav Nutr Phys Act.

[34] Alkhaldi G, Modrow K, Hamilton F, Pal K, Ross J, Murray E (2017). Promoting engagement with a digital health intervention (HeLP-Diabetes) using email and text message prompts: mixed-methods study. Interact J Med Res.

[35] Pal K, Dack C, Ross J, Michie S, May C, Stevenson F, Farmer A, Yardley L, Barnard M, Murray E (2018). Digital health interventions for adults with type 2 diabetes: qualitative study of patient perspectives on diabetes self-management education and support. J Med Internet Res.

[36] Webb TL, Joseph J, Yardley L, Michie S (2010). Using the internet to promote health behavior change: a systematic review and meta-analysis of the impact of theoretical basis, use of behavior change techniques, and mode of delivery on efficacy. J Med Internet Res.

[37] Brouwer W, Kroeze W, Crutzen R, de Nooijer J, de Vries NK, Brug J, Oenema A (2011). Which intervention characteristics are related to more exposure to internet-delivered healthy lifestyle promotion interventions? A systematic review. J Med Internet Res.

[38] Sharpe EE, Karasouli E, Meyer C (2017). Examining factors of engagement with digital interventions for weight management: rapid review. JMIR Res Protoc.

[39] Gan DZ, McGillivray L, Larsen ME, Christensen H, Torok M (2022). Technology-supported strategies for promoting user engagement with digital mental health interventions: a systematic review. Digit Health.

[40] Milward J, Drummond C, Fincham-Campbell S, Deluca P (2018). What makes online substance-use interventions engaging? A systematic review and narrative synthesis. Digit Health.

[41] Cochrane handbook for systematic reviews of interventions version 5.1.0. 2011. The Cochrane Collaboration.

[42] Grady A, Yoong SL, Wolfenden L, Wyse R, Sutherland R, Nathan N, Mclaughlin M, Delaney T, Hodder R (2018). The effectiveness of strategies to improve user engagement with digital health interventions to improve risk factors for chronic disease: a systematic review. National Institute for Health and Care Research.

[43] Lefebvre C, Manheimer E, Glanville J, Higgins JP, Green S (2011). Searching for studies. Cochrane Handbook for Systematic Reviews of Interventions Version 5.

[44] Waters E, de Silva-Sanigorski A, Hall BJ, Brown T, Campbell K, Gao Y, Armstrong R, Prosser L, Summerbell CD (2011). Interventions for preventing obesity in children. Cochrane Database Syst Rev.

[45] Wolfenden L, Barnes C, Jones J, Finch M, Wyse R, Kingsland M, Tzelepis F, Grady A, Hodder RK, Booth D, Yoong SL (2020). Strategies to improve the implementation of healthy eating, physical activity and obesity prevention policies, practices or programmes within childcare services. Cochrane Database Syst Rev.

[46] Wolfenden L, Nathan NK, Sutherland R, Yoong SL, Hodder RK, Wyse RJ, Delaney T, Grady A, Fielding A, Tzelepis F, Clinton-McHarg T, Parmenter B, Butler P, Wiggers J, Bauman A, Milat A, Booth D, Williams CM (2017). Strategies for enhancing the implementation of school-based policies or practices targeting risk factors for chronic disease. Cochrane Database Syst Rev.

[47] (2019). Covidence Systematic review software. Veritas Health Innovation.

[48] Page MJ, McKenzie JE, Bossuyt PM, Boutron I, Hoffmann TC, Mulrow CD, Shamseer L, Tetzlaff JM, Akl EA, Brennan SE, Chou R, Glanville J, Grimshaw JM, Hróbjartsson A, Lalu MM, Li T, Loder EW, Mayo-Wilson E, McDonald S, McGuinness LA, Stewart LA, Thomas J, Tricco AC, Welch VA, Whiting P, Moher D (2021). The PRISMA 2020 statement: an updated guideline for reporting systematic reviews. Syst Rev.

[49] (2011). Data Extraction and Assessment Template. The Cochrane Public Health Group.

[50] McCann L, McMillan KA, Pugh G (2019). Digital interventions to support adolescents and young adults with cancer: systematic review. JMIR Cancer.

[51] Higgins JP, Altman DG, Gøtzsche PC, Jüni P, Moher D, Oxman AD, Savovic J, Schulz KF, Weeks L, Sterne JA, Cochrane Bias Methods Group, Cochrane Statistical Methods Group (2011). The Cochrane Collaboration's tool for assessing risk of bias in randomised trials. BMJ.

[52] Wolfenden L, McCrabb S, Barnes C, O'Brien K, Ng K, Nathan N, Sutherland R, Hodder RK, Tzelepis F, Nolan E, Williams CM, Yoong SL (2022). Strategies for enhancing the implementation of school-based policies or practices targeting diet, physical activity, obesity, tobacco or alcohol use. Cochrane Database Syst Rev.

[53] Hattle M, Burke DL, Trikalinos T, Schmid CH, Chen Y, Jackson D, Riley RD (2022). Multivariate meta-analysis of multiple outcomes: characteristics and predictors of borrowing of strength from Cochrane reviews. Syst Rev.

[54] Tanner-Smith EE, Tipton E, Polanin JR (2016). Handling Complex Meta-analytic Data Structures Using Robust Variance Estimates: a Tutorial in R. J Dev Life Course Criminol.

[55] West DS, Krukowski RA, Finkelstein EA, Stansbury ML, Ogden DE, Monroe CM, Carpenter CA, Naud S, Harvey JR (2020). Adding financial incentives to online group-based behavioral weight control: an RCT. Am J Prev Med.

[56] Fisher Z, Tipton E, Zhipeng H (2017). Robumeta: robust variance meta-regression. R package version.

[57] Cohen J (1988). Statistical Power Analysis for the Behavioral Sciences. 2nd edition.

[58] Campbell M, McKenzie JE, Sowden A, Katikireddi SV, Brennan SE, Ellis S, Hartmann-Boyce J, Ryan R, Shepperd S, Thomas J, Welch V, Thomson H (2020). Synthesis without meta-analysis (SWiM) in systematic reviews: reporting guideline. BMJ.

[59] Yoong S, Lum M, Wolfenden L, Jackson J, Barnes C, Hall AE, McCrabb S, Pearson N, Lane C, Jones JZ, Dinour L, McDonnell T, Booth D, Grady A (2023). Healthy eating interventions delivered in early childhood education and care settings for improving the diet of children aged six months to six years. Cochrane Database Syst Rev.

[60] Alley S, Jennings C, Persaud N, Plotnikoff RC, Horsley M, Vandelanotte C (2014). Do personally tailored videos in a web-based physical activity intervention lead to higher attention and recall? - An eye-tracking study. Front Public Health.

[61] Alley S, Jennings C, Plotnikoff RC, Vandelanotte C (2016). Web-based video-coaching to assist an automated computer-tailored physical activity intervention for inactive adults: a randomized controlled trial. J Med Internet Res.

[62] Blanson Henkemans OA, van der Boog PJ, Lindenberg J, van der Mast CA, Neerincx MA, Zwetsloot-Schonk BJ (2009). An online lifestyle diary with a persuasive computer assistant providing feedback on self-management. Technol Health Care.

[63] Brindal E, Freyne J, Saunders I, Berkovsky S, Smith G, Noakes M (2012). Features predicting weight loss in overweight or obese participants in a web-based intervention: randomized trial. J Med Internet Res.

[64] Brindal E, Hendrie GA, Freyne J, Noakes M (2019). A mobile phone app designed to support weight loss maintenance and well-being (MotiMate): randomized controlled trial. JMIR Mhealth Uhealth.

[65] Chai LK, Collins CE, May C, Brown LJ, Ashman A, Burrows TL (2021). Fidelity and acceptability of a family-focused technology-based telehealth nutrition intervention for child weight management. J Telemed Telecare.

[66] Couper MP, Alexander GL, Zhang N, Little RJ, Maddy N, Nowak MA, McClure JB, Calvi JJ, Rolnick SJ, Stopponi MA, Cole Johnson C (2010). Engagement and retention: measuring breadth and depth of participant use of an online intervention. J Med Internet Res.

[67] Dennison L, Morrison L, Lloyd S, Phillips D, Stuart B, Williams S, Bradbury K, Roderick P, Murray E, Michie S, Little P, Yardley L (2014). Does brief telephone support improve engagement with a web-based weight management intervention? Randomized controlled trial. J Med Internet Res.

[68] Ellingson LD, Lansing JE, DeShaw KJ, Peyer KL, Bai Y, Perez M, Phillips LA, Welk GJ (2019). Evaluating motivational interviewing and habit formation to enhance the effect of activity trackers on healthy adults' activity levels: randomized intervention. JMIR Mhealth Uhealth.

[69] Gabriele JM, Carpenter BD, Tate DF, Fisher EB (2011). Directive and nondirective e-coach support for weight loss in overweight adults. Ann Behav Med.

[70] Hutchesson MJ, Tan CY, Morgan P, Callister R, Collins C (2016). Enhancement of self-monitoring in a web-based weight loss program by extra individualized feedback and reminders: randomized trial. J Med Internet Res.

[71] Kliemann N, Croker H, Johnson F, Beeken RJ (2019). Development of the top tips habit-based weight loss app and preliminary indications of its usage, effectiveness, and acceptability: mixed-methods pilot study. JMIR Mhealth Uhealth.

[72] LaRose JG, Tate DF, Lanoye A, Fava JL, Jelalian E, Blumenthal M, Caccavale LJ, Wing RR (2019). Adapting evidence-based behavioral weight loss programs for emerging adults: a pilot randomized controlled trial. J Health Psychol.

[73] Mailey EL, Huberty J, Irwin BC (2016). Feasibility and effectiveness of a web-based physical activity intervention for working mothers. J Phys Act Health.

[74] Micco N, Gold B, Buzzell P, Leonard H, Pintauro S, Harvey-Berino J (2007). Minimal in-person support as an adjunct to internet obesity treatment. Ann Behav Med.

[75] Monroe CM, Geraci M, Larsen CA, West DS (2019). Feasibility and efficacy of a novel technology-based approach to harness social networks for weight loss: the NETworks pilot randomized controlled trial. Obes Sci Pract.

[76] Napolitano MA, Hayes S, Bennett GG, Ives AK, Foster GD (2013). Using Facebook and text messaging to deliver a weight loss program to college students. Obesity (Silver Spring).

[77] Newton RL, Marker AM, Allen HR, Machtmes R, Han H, Johnson WD, Schuna JM, Broyles ST, Tudor-Locke C, Church TS (2014). Parent-targeted mobile phone intervention to increase physical activity in sedentary children: randomized pilot trial. JMIR Mhealth Uhealth.

[78] Omran J, Trinh L, Arbour-Nicitopoulos KP, Mitchell MS, Faulkner GE (2018). Do incentives promote action planning in a web-based walking intervention?. Am J Health Behav.

[79] Patel ML, Hopkins CM, Brooks TL, Bennett GG (2019). Comparing self-monitoring strategies for weight loss in a smartphone app: randomized controlled trial. JMIR Mhealth Uhealth.

[80] Ross KM, Wing RR (2016). Impact of newer self-monitoring technology and brief phone-based intervention on weight loss: a randomized pilot study. Obesity (Silver Spring).

[81] Shaw RJ (2012). A mobile health intervention to sustain recent weight loss. Duke Dissertations.

[82] Soetens KC, Vandelanotte C, de Vries H, Mummery KW (2014). Using online computer tailoring to promote physical activity: a randomized trial of text, video, and combined intervention delivery modes. J Health Commun.

[83] Sze YY, Daniel TO, Kilanowski CK, Collins RL, Epstein LH (2015). Web-based and mobile delivery of an episodic future thinking intervention for overweight and obese families: a feasibility study. JMIR Mhealth Uhealth.

[84] Tsai CC, Lee G, Raab F, Norman GJ, Sohn T, Griswold WG, Patrick K (2007). Usability and feasibility of PmEB: a mobile phone application for monitoring real time caloric balance. Mob Netw Appl.

[85] Vandelanotte C, Kolt GS, Caperchione CM, Savage TN, Rosenkranz RR, Maeder AJ, Van Itallie A, Tague R, Oldmeadow C, Mummery WK, Duncan MJ (2017). Effectiveness of a web 2.0 intervention to increase physical activity in real-world settings: randomized ecological trial. J Med Internet Res.

[86] Vandelanotte C, Duncan MJ, Maher CA, Schoeppe S, Rebar AL, Power DA, Short CE, Doran CM, Hayman MJ, Alley SJ (2018). The effectiveness of a web-based computer-tailored physical activity intervention using Fitbit activity trackers: randomized trial. J Med Internet Res.

[87] Walthouwer MJ, Oenema A, Lechner L, de Vries H (2015). Comparing a video and text version of a web-based computer-tailored intervention for obesity prevention: a randomized controlled trial. J Med Internet Res.

[88] Wang JB, Cadmus-Bertram LA, Natarajan L, White MM, Madanat H, Nichols JF, Ayala GX, Pierce JP (2015). Wearable sensor/device (Fitbit One) and SMS text-messaging prompts to increase physical activity in overweight and obese adults: a randomized controlled trial. Telemed J E Health.

[89] West DS, Harvey JR, Krukowski RA, Prewitt TE, Priest J, Ashikaga T (2016). Do individual, online motivational interviewing chat sessions enhance weight loss in a group-based, online weight control program?. Obesity (Silver Spring).

[90] Alley SJ, Schoeppe S, To QG, Parkinson L, van Uffelen J, Hunt S, Duncan MJ, Schneiders A, Vandelanotte C (2023). Engagement, acceptability, usability and satisfaction with active for life, a computer-tailored web-based physical activity intervention using Fitbit in older adults. Int J Behav Nutr Phys Act.

[91] Beleigoli A, Andrade AQ, Diniz MD, Ribeiro AL (2020). Personalized web-based weight loss behavior change program with and without dietitian online coaching for adults with overweight and obesity: randomized controlled trial. J Med Internet Res.

[92] Butryn ML, Martinelli MK, Crane NT, Godfrey K, Roberts SR, Zhang F, Forman EM (2020). Counselor surveillance of digital self-monitoring data: a pilot randomized controlled trial. Obesity (Silver Spring).

[93] Eisenhauer CM, Brito F, Kupzyk K, Yoder A, Almeida F, Beller RJ, Miller J, Hageman PA (2021). Mobile health assisted self-monitoring is acceptable for supporting weight loss in rural men: a pragmatic randomized controlled feasibility trial. BMC Public Health.

[94] Forman EM, Goldstein SP, Crochiere RJ, Butryn ML, Juarascio AS, Zhang F, Foster GD (2019). Randomized controlled trial of OnTrack, a just-in-time adaptive intervention designed to enhance weight loss. Transl Behav Med.

[95] Granet J, Peyrusqué E, Ruiz F, Buckinx F, Abdelkader LB, Dang-Vu TT, Sirois MJ, Gouin JP, Pageaux B, Aubertin-Leheudre M (2023). Online physical exercise intervention in older adults during lockdown: can we improve the recipe?. Aging Clin Exp Res.

[96] Haslam RL, Baldwin JN, Pezdirc K, Truby H, Attia J, Hutchesson MJ, Burrows T, Callister R, Hides L, Bonevski B, Kerr DA, Kirkpatrick SI, Rollo ME, McCaffrey TA, Collins CE (2023). Efficacy of technology-based personalised feedback on diet quality in young Australian adults: results for the advice, ideas and motivation for my eating (Aim4Me) randomised controlled trial. Public Health Nutr.

[97] Jin Q, Boyce TW, Kang H, Nervi L, Sussman AL, Guest DD (2021). Acceptability of phone calls and texts to promote healthy behaviors among Spanish-speaking Hispanics. Hisp J Behav Sci.

[98] LaRose JG, Gorin AA, Fava JL, Bean MK, Lanoye A, Robinson E, Carey K (2020). Using motivational interviewing to enhance emerging adults' engagement in weight loss: the Live Well RVA pilot randomized clinical trial. Obes Sci Pract.

[99] LaRose JG, Leahey TM, Lanoye A, Reading J, Wing RR (2020). A secondary data analysis examining young adults' performance in an internet weight loss program with financial incentives. Obesity (Silver Spring).

[100] Levin ME, Krafft J, Seifert S, Lillis J (2022). Tracking valued and avoidant functions with health behaviors: a randomized controlled trial of the acceptance and commitment therapy Matrix mobile app. Behav Modif.

[101] Pischke CR, Voelcker-Rehage C, Ratz T, Peters M, Buck C, Meyer J, von Holdt K, Lippke S (2022). Web-based versus print-based physical activity intervention for community-dwelling older adults: crossover randomized trial. JMIR Mhealth Uhealth.

[102] Schoeppe S, Duncan MJ, Plotnikoff RC, Mummery WK, Rebar A, Alley S, To Q, Short CE, Vandelanotte C (2022). Acceptability, usefulness, and satisfaction with a web-based video-tailored physical activity intervention: the TaylorActive randomized controlled trial. J Sport Health Sci.

[103] Kolt GS, Rosenkranz RR, Vandelanotte C, Caperchione CM, Maeder AJ, Tague R, Savage TN, Van IA, Mummery WK, Oldmeadow C, Duncan MJ (2017). Using Web 2.0 applications to promote health-related physical activity: findings from the WALK 2.0 randomised controlled trial. Br J Sports Med.

[104] Pullen CH, Hageman PA, Boeckner L, Walker SN, Oberdorfer MK (2008). Feasibility of Internet-delivered weight loss interventions among rural women ages 50-69. J Geriatr Phys Ther.

[105] Fanning J, Roberts S, Hillman CH, Mullen SP, Ritterband L, McAuley E (2017). A smartphone "app"-delivered randomized factorial trial targeting physical activity in adults. J Behav Med.

[106] Nour M, Chen J, Allman-Farinelli M (2019). Young adults' engagement with a self-monitoring app for vegetable intake and the impact of social media and gamification: feasibility study. JMIR Form Res.

[107] Nuijten R, Van GP, Kaymak U, Simons M, Kemperman A, Van DB (2019). Evaluation of the impact of extrinsic rewards on user engagement in a health promotion context. Proceedings of the 41st Annual International Conference of the IEEE Engineering in Medicine and Biology Society.

[108] Kwan M, Faulkner G, Bray S (2013). Evaluation of active transition, a website-delivered physical activity intervention for university students: pilot study. JMIR Res Protoc.

[109] Liao J, Xiao HY, Li X, Sun SX, Liu S, Yang YJ, Xu DR (2020). A social group-based information-motivation-behavior skill intervention to promote acceptability and adoption of wearable activity trackers among middle-aged and older adults: cluster randomized controlled trial. JMIR Mhealth Uhealth.

[110] Guagliano JM, Brown HE, Coombes E, Hughes C, Jones AP, Morton KL, Wilson EC, van Sluijs EM (2019). The development and feasibility of a randomised family-based physical activity promotion intervention: the Families Reporting Every Step to Health (FRESH) study. Pilot Feasibility Stud.

[111] Edney S, Ryan JC, Olds T, Monroe C, Fraysse F, Vandelanotte C, Plotnikoff R, Curtis R, Maher C (2019). User engagement and attrition in an app-based physical activity intervention: secondary analysis of a randomized controlled trial. J Med Internet Res.

[112] Vandelanotte C, Short CE, Plotnikoff RC, Rebar A, Alley S, Schoeppe S, Canoy DF, Hooker C, Power D, Oldmeadow C, Leigh L, To Q, Mummery WK, Duncan MJ (2021). Are web-based personally tailored physical activity videos more effective than personally tailored text-based interventions? Results from the three-arm randomised controlled TaylorActive trial. Br J Sports Med.

[113] Wang J (2014). A wearable sensor (Fitbit One) and text-messaging to promote physical activity and participants' level of engagement (a randomized controlled feasibility trial). UC San Diego.

[114] Gabriele JM (2009). Effects of nondirective and directive support on weight loss in an e-counseling intervention. Retrospective Theses and Dissertations.

[115] Alley SJ, van Uffelen J, Schoeppe S, Parkinson L, Hunt S, Power D, Waterman N, Waterman C, To QG, Duncan MJ, Schneiders A, Vandelanotte C (2022). The effectiveness of a computer-tailored web-based physical activity intervention using Fitbit activity trackers in older adults (active for life): randomized controlled trial. J Med Internet Res.

[116] Chai LK, Collins CE, May C, Ashman A, Holder C, Brown LJ, Burrows TL (2021). Feasibility and efficacy of a web-based family telehealth nutrition intervention to improve child weight status and dietary intake: a pilot randomised controlled trial. J Telemed Telecare.

[117] Edney SM, Olds TS, Ryan JC, Vandelanotte C, Plotnikoff RC, Curtis RG, Maher CA (2020). A social networking and gamified app to increase physical activity: cluster RCT. Am J Prev Med.

[118] Collins CE, Morgan PJ, Hutchesson MJ, Callister R (2013). Efficacy of standard versus enhanced features in a Web-based commercial weight-loss program for obese adults, part 2: randomized controlled trial. J Med Internet Res.

[119] Collins CE, Morgan PJ, Jones P, Fletcher K, Martin J, Aguiar EJ, Lucas A, Neve MJ, Callister R (2012). A 12-week commercial web-based weight-loss program for overweight and obese adults: randomized controlled trial comparing basic versus enhanced features. J Med Internet Res.

[120] Leahey TM, Subak LL, Fava J, Schembri M, Thomas G, Xu X, Krupel K, Kent K, Boguszewski K, Kumar R, Weinberg B, Wing R (2015). Benefits of adding small financial incentives or optional group meetings to a web-based statewide obesity initiative. Obesity (Silver Spring).

[121] Vandelanotte C, Duncan M, Mummery K (2011). Initial effectiveness and acceptability of a video-tailored physical activity intervention. J Sci Med Sport.

[122] Walthouwer MJ, Oenema A, Lechner L, de Vries H (2015). Use and effectiveness of a video- and text-driven web-based computer-tailored intervention: randomized controlled trial. J Med Internet Res.

[123] Maher CA, Lewis LK, Ferrar K, Marshall S, De Bourdeaudhuij I, Vandelanotte C (2014). Are health behavior change interventions that use online social networks effective? A systematic review. J Med Internet Res.

[124] Jakob R, Harperink S, Rudolf AM, Fleisch E, Haug S, Mair JL, Salamanca-Sanabria A, Kowatsch T (2022). Factors influencing adherence to mHealth apps for prevention or management of noncommunicable diseases: systematic review. J Med Internet Res.

[125] van Genugten L, Dusseldorp E, Webb TL, van Empelen P (2016). Which combinations of techniques and modes of delivery in internet-based interventions effectively change health behavior? A meta-analysis. J Med Internet Res.

[126] Eysenbach G, CONSORT-EHEALTH Group (2011). CONSORT-EHEALTH: improving and standardizing evaluation reports of web-based and mobile health interventions. J Med Internet Res.

