# Supplementary material for: The Effectiveness of Strategies to Improve User Engagement With Digital Health Interventions Targeting Nutrition, Physical Activity, and Overweight and Obesity: Systematic Review and Meta-Analysis
Source: J Med Internet Res. 2023 Dec 19;25:e47987. doi: 10.2196/47987 (PMC10762625; doi:10.2196/47987)
Supplement: Multimedia Appendix 1 [file jmir_v25i1e47987_app1.docx]

**Multimedia Appendix 1. Search terms**

Database(s): **Ovid MEDLINE(R) Epub Ahead of Print, In-Process & Other Non-Indexed Citations, Ovid MEDLINE(R) Daily and Ovid MEDLINE(R)**1946 to Present 
Search Strategy:

| **#** | **Searches** |
| --- | --- |
| 1 | (phone adj app*).mp. |
| 2 | Cell Phone/ or Mobile Applications/ or SMARTPHONE/ or (mobile device* or smartphone*).mp. |
| 3 | Virtual Reality/ |
| 4 | ONLINE SYSTEMS/ or online.mp. |
| 5 | internet/ or blogging/ or social media/ |
| 6 | ((internet or web) adj based).mp. |
| 7 | (world wide web or WWW or website*).mp. |
| 8 | Electronic Mail/ or (e-mail* or email*).mp. |
| 9 | ((mobile or cellular or cell or smart) adj (phone* or telephone*)).mp. |
| 10 | Telemedicine/ or (e-health or ehealth or electronic health).mp. |
| 11 | (m-health or mhealth or mobile health).mp. |
| 12 | ((digital health or digital) adj intervention*).mp. |
| 13 | (interactive adj ((health adj communicat*) or video* or technolog* or multimedia)).mp. |
| 14 | ((chat adj room*) or chatroom*).mp. |
| 15 | Fitness Trackers/ or activity tracker*.mp. |
| 16 | Computer Communication Networks/ |
| 17 | medical informatics/ or information technology/ |
| 18 | Therapy, Computer-Assisted/ |
| 19 | User-Computer Interface/ or human computer interaction*.mp. |
| 20 | 1 or 2 or 3 or 4 or 5 or 6 or 7 or 8 or 9 or 10 or 11 or 12 or 13 or 14 or 15 or 16 or 17 or 18 or 19 |
| 21 | Engag*.mp. |
| 22 | attrition.mp. or Patient Dropouts/ |
| 23 | adher*.mp. |
| 24 | COMPLIANCE/ or PATIENT COMPLIANCE/ |
| 25 | Patient Participation/ |
| 26 | ((intervention or online or web*) adj3 (adopt* or uptake or retention or maintenance or efficacy or us* or reach* or participat* or exposure)).mp. |
| 27 | (process adj (metric* or evaluation)).mp. |
| 28 | login*.mp. |
| 29 | (page* adj3 view*).mp. |
| 30 | ((module* or session*) adj complet*).mp. |
| 31 | (visit* adj3 website*).mp. |
| 32 | implement*.mp. |
| 33 | feasibil*.mp. |
| 34 | time spent online.mp. |
| 35 | (user adj3 (engag* or experience* or enjoyment or attention or interest or affect or immersion or involvement)).mp. |
| 36 | Subjective experience*.mp. |
| 37 | usability.mp. |
| 38 | 21 or 22 or 23 or 24 or 25 or 26 or 27 or 28 or 29 or 30 or 31 or 32 or 33 or 34 or 35 or 36 or 37 |
| 39 | exp Exercise/ |
| 40 | physical activit*.mp. |
| 41 | physical inactivit*.mp. |
| 42 | (physical education and training).mp. |
| 43 | "Physical Education and Training"/ |
| 44 | Physical Fitness/ |
| 45 | sedentary.mp. |
| 46 | exp Leisure Activities/ |
| 47 | exp Sports/ |
| 48 | (exercise* adj aerobic*).mp. |
| 49 | sport*.mp. |
| 50 | ((lifestyle* or life style*) adj5 activ*).mp. |
| 51 | Motor Activity/ |
| 52 | or/39-51 |
| 53 | exp Diet/ |
| 54 | healthy eating.mp. |
| 55 | Fruit/ or fruit*.mp. |
| 56 | Vegetable/ or vegetable*.mp. |
| 57 | (canteen* or cafeteria*).mp. |
| 58 | Food Services/ or food service*.mp. |
| 59 | calorie*.mp. |
| 60 | Energy Intake/ |
| 61 | energy density.mp. |
| 62 | Feeding Behavior/ or Eating/ or feeding behavio*.mp. |
| 63 | dietary intake.mp. |
| 64 | food habits.mp. |
| 65 | Food/ |
| 66 | Dietary Fats, Unsaturated/ or Dietary Fats/ |
| 67 | ((feeding or food or nutrition*) adj program*).mp. |
| 68 | Nutritional Status/ |
| 69 | PARENTERAL NUTRITION/ or CHILD NUTRITION DISORDERS/ or nutrition*.mp. or NUTRITION ASSESSMENT/ |
| 70 | meals/ or breakfast/ or lunch/ or snacks/ |
| 71 | MENU PLANNING/ or menu*.mp. |
| 72 | (food adj purchas*).mp. |
| 73 | Carbonated Beverages/ or (soft drink or soda).mp. |
| 74 | ((sweetened or sugar-sweetened) adj drink*).mp. |
| 75 | or/53-74 |
| 76 | SMOKING PREVENTION/ or SMOKING CESSATION/ or exp SMOKING/ |
| 77 | "Tobacco Use Cessation"/ |
| 78 | smok*.mp. |
| 79 | Nicotine/ |
| 80 | Tobacco/ or "Tobacco Use"/ |
| 81 | ((ceas* or cess* or prevent* or stop* or quit* or abstin* or abstain* or reduc*) adj5 (tobacco or nicotine)).mp. |
| 82 | "Tobacco Use Disorder"/ |
| 83 | or/76-82 |
| 84 | Health Promotion/ or Health Education/ or Health Behavior/ or Health Knowledge, Attitudes, Practice/ or health behav*.mp. |
| 85 | Life Style/ |
| 86 | Primary Prevention/ |
| 87 | Secondary Prevention/ |
| 88 | prevent* care.mp. |
| 89 | or/84-88 |
| 90 | Obesity/ or Obesity, Morbid/ or Pediatric obesity/ or obes*.mp. |
| 91 | Weight Gain/ |
| 92 | Weight Loss/ |
| 93 | Body weight/ or weight manag*.mp. |
| 94 | weight control.mp. |
| 95 | Overweight/ or (over weight or overeat* or over eat*).mp. |
| 96 | weight change*.mp. |
| 97 | weight status.mp. |
| 98 | Body Mass Index/ or bmi.mp. |
| 99 | or/90-98 |
| 100 | alcohol drinking/ or binge drinking/ |
| 101 | alcohol*.mp. |
| 102 | alcoholic intoxication/ or alcoholism/ |
| 103 | drink*.mp. |
| 104 | drunk.mp. |
| 105 | intoxicat*.mp. |
| 106 | or/100-105 |
| 107 | 52 or 75 or 83 or 89 or 99 or 106 |
| 108 | (prompt* or reminder* or (push adj notification*)).mp. |
| 109 | ((chat adj room*) or chatroom*).mp. |
| 110 | ((bulletin adj board*) or (message adj board*)).mp. |
| 111 | ((online adj social adj network*) or (social adj technolog*) or (social adj network* adj site*)).mp. |
| 112 | (tailor* or personali* or relevan* or individuali*).mp. |
| 113 | feedback.mp. |
| 114 | (Gamification or (gam* adj strateg*)).mp. |
| 115 | (Incentive* or reinforcement* or reward*).mp. |
| 116 | (goal adj setting).mp. |
| 117 | monitor*.mp. |
| 118 | ((leader adj board) or leaderboard or (progress adj report) or (progress adj chart)).mp. |
| 119 | (multimedia messag* or MMS).mp. |
| 120 | (text message* or short messag* service* or SMS).mp. |
| 121 | or/108-120 |
| 122 | Randomized Controlled Trial/ |
| 123 | clinical trial/ or controlled clinical trial/ |
| 124 | random allocation/ |
| 125 | Double-Blind Method/ |
| 126 | Single-Blind Method/ |
| 127 | placebos/ |
| 128 | Research Design/ |
| 129 | Evaluation Studies/ |
| 130 | Comparative Study/ |
| 131 | Cross-Over Studies/ |
| 132 | placebo*.tw. |
| 133 | random*.tw. |
| 134 | control*.tw. |
| 135 | comparison group*.tw. |
| 136 | (quasiexperimental or quasi experimental or pseudo experimental).tw. |
| 137 | or/122-136 |
| 138 | 20 and 38 and 107 and 121 and 137 |
| 139 | animals/ not (humans/ and animals/) |
| 140 | 138 not 139 |
| **141** | **limit 140 to english language** |

Database(s): **Embase**1947 to present 
Search Strategy:

| **#** | **Searches** |
| --- | --- |
| 1 | (phone adj app*).mp. |
| 2 | mobile application/ or mobile phone/ or smartphone/ or (smartphone* or mobile device*).mp. |
| 3 | virtual reality/ |
| 4 | online system/ or online.mp. |
| 5 | internet/ or blogging/ or social media/ |
| 6 | ((internet or web) adj based).mp. |
| 7 | (world wide web or WWW or website*).mp. |
| 8 | e-mail/ or email*.mp. |
| 9 | ((mobile or cellular or cell or smart) adj (phone* or telephone*)).mp. |
| 10 | Telemedicine/ or (e-health or ehealth or electronic health).mp. |
| 11 | (m-health or mhealth or mobile health).mp. |
| 12 | ((digital health or digital) adj intervention*).mp. |
| 13 | (interactive adj ((health adj communicat*) or video* or technolog* or multimedia)).mp. |
| 14 | ((chat adj room*) or chatroom*).mp. |
| 15 | activity tracker/ or activity tracker*.mp. |
| 16 | computer network/ |
| 17 | medical informatics/ or information technology/ |
| 18 | computer assisted therapy/ |
| 19 | human computer interaction/ or computer interface/ |
| 20 | or/1-19 |
| 21 | Engag*.mp. |
| 22 | attrition.mp. or Patient Dropout/ |
| 23 | adher*.mp. |
| 24 | patient compliance/ |
| 25 | patient participation/ |
| 26 | ((intervention or online or web*) adj3 (adopt* or uptake or retention or maintenance or efficacy or us* or reach* or participat* or exposure)).mp. |
| 27 | (process adj (metric* or evaluation)).mp. |
| 28 | login*.mp. |
| 29 | (page* adj3 view*).mp. |
| 30 | ((module* or session*) adj complet*).mp. |
| 31 | (visit* adj3 website*).mp. |
| 32 | implement*.mp. |
| 33 | feasibil*.mp. |
| 34 | time spent online.mp. |
| 35 | (user adj3 (engag* or experience* or enjoyment or attention or interest or affect or immersion or involvement)).mp. |
| 36 | Subjective experience*.mp. |
| 37 | usability.mp. |
| 38 | or/21-37 |
| 39 | exp exercise/ |
| 40 | physical activity/ |
| 41 | physical inactivity/ |
| 42 | "Physical Education and Training".mp. or physical education/ |
| 43 | Physical Fitness.mp. or fitness/ |
| 44 | sedentary.mp. |
| 45 | Leisure Activities.mp. or leisure/ |
| 46 | exp sport/ |
| 47 | (exercise* adj aerobic*).mp. |
| 48 | sport*.mp. |
| 49 | ((lifestyle* or life style*) adj5 activ*).mp. |
| 50 | motor activity/ |
| 51 | or/39-50 |
| 52 | exp diet/ |
| 53 | healthy eating.mp. |
| 54 | Fruit*.mp. or fruit/ |
| 55 | vegetable/ or vegetable*.mp. |
| 56 | (canteen* or cafeteria*).mp. |
| 57 | catering service/ or food service*.mp. |
| 58 | calorie*.mp. |
| 59 | Energy Intake.mp. or caloric intake/ |
| 60 | energy density.mp. |
| 61 | feeding behavior/ or eating/ or feeding behavio*.mp. |
| 62 | dietary intake/ |
| 63 | food habits.mp. |
| 64 | food/ |
| 65 | fat intake/ or dietary fats.mp. |
| 66 | ((feeding or food or nutrition*) adj program*).mp. |
| 67 | nutritional status/ |
| 68 | total parenteral nutrition/ or child nutrition/ or nutrition*.mp. |
| 69 | meal/ or (breakfast or meal* or snack* or lunch).mp. |
| 70 | menu*.mp. |
| 71 | (food adj purchas*).mp. |
| 72 | Carbonated Beverage/ or (soft drink or soda).mp. |
| 73 | ((sweetened or sugar-sweetened) adj drink*).mp. |
| 74 | or/52-73 |
| 75 | smoking cessation/ or exp smoking/ |
| 76 | smok*.mp. |
| 77 | nicotine/ |
| 78 | tobacco/ or "tobacco use"/ |
| 79 | ((ceas* or cess* or prevent* or stop* or quit* or abstin* or abstain* or reduc*) adj5 (tobacco or nicotine)).mp. |
| 80 | tobacco dependence/ |
| 81 | or/75-80 |
| 82 | health education/ or health behavior/ or health promotion/ or "attitude to health"/ or health behav*.mp. |
| 83 | lifestyle/ |
| 84 | primary prevention/ |
| 85 | secondary prevention/ |
| 86 | prevent* care.mp. |
| 87 | or/82-86 |
| 88 | Obesity/ or Obesity, Morbid/ or childhood obesity/ or obes*.mp. |
| 89 | weight gain/ |
| 90 | Weight Loss.mp. or weight reduction/ |
| 91 | Body weight/ or weight manag*.mp. |
| 92 | weight control.mp. |
| 93 | Overweight/ or (over weight or overeat* or over eat*).mp. |
| 94 | weight change*.mp. |
| 95 | weight status.mp. |
| 96 | Body Mass Index/ or bmi.mp. |
| 97 | or/88-96 |
| 98 | drinking behavior/ |
| 99 | binge drinking/ |
| 100 | alcohol*.mp. |
| 101 | alcohol intoxication/ or alcoholism/ |
| 102 | drink*.mp. |
| 103 | drunk.mp. |
| 104 | intoxicat*.mp. |
| 105 | or/98-104 |
| 106 | 51 or 74 or 81 or 87 or 97 or 105 |
| 107 | (prompt* or reminder* or (push adj notification*)).mp. |
| 108 | ((chat adj room*) or chatroom*).mp. |
| 109 | ((bulletin adj board*) or (message adj board*)).mp. |
| 110 | ((online adj social adj network*) or (social adj technolog*) or (social adj network* adj site*)).mp. |
| 111 | (tailor* or personali* or relevan* or individuali*).mp. |
| 112 | feedback.mp. |
| 113 | (Gamification or (gam* adj strateg*)).mp. |
| 114 | (Incentive* or reinforcement* or reward*).mp. |
| 115 | (goal adj setting).mp. |
| 116 | monitor*.mp. |
| 117 | ((leader adj board) or leaderboard or (progress adj report) or (progress adj chart)).mp. |
| 118 | (multimedia messag* or MMS).mp. |
| 119 | (text message* or short messag* service* or SMS).mp. |
| 120 | or/107-119 |
| 121 | randomized controlled trial/ |
| 122 | clinical trial/ or controlled clinical trial/ |
| 123 | randomization/ |
| 124 | double blind procedure/ |
| 125 | single blind procedure/ |
| 126 | placebo/ |
| 127 | Research Design.mp. |
| 128 | evaluation study/ |
| 129 | comparative study/ |
| 130 | crossover procedure/ |
| 131 | placebo*.tw. |
| 132 | random*.tw. |
| 133 | control*.tw. |
| 134 | comparison group*.tw. |
| 135 | (quasiexperimental or quasi experimental or pseudo experimental).tw. |
| 136 | or/121-135 |
| 137 | 20 and 38 and 106 and 120 and 136 |
| **138** | **limit 137 to (human and english language)** |

Database(s): **PsycINFO**1806 to June Week 3 2018 
Search Strategy:

| **#** | **Searches** |
| --- | --- |
| 1 | (phone adj app*).mp. |
| 2 | cellular phones/ or Mobile Devices/ or (mobile device* or smartphone* or mobile application*).mp. |
| 3 | Virtual Reality/ |
| 4 | online.mp. or ONLINE SOCIAL NETWORKS/ |
| 5 | internet/ or blog/ or social media/ |
| 6 | ((internet or web) adj based).mp. |
| 7 | (world wide web or WWW or website*).mp. |
| 8 | Computer Mediated Communication/ or (e-mail* or email*).mp. |
| 9 | ((mobile or cellular or cell or smart) adj (phone* or telephone*)).mp. |
| 10 | Telemedicine/ or (e-health or ehealth or electronic health).mp. |
| 11 | (m-health or mhealth or mobile health).mp. |
| 12 | ((digital health or digital) adj intervention*).mp. |
| 13 | (interactive adj ((health adj communicat*) or video* or technolog* or multimedia)).mp. |
| 14 | ((chat adj room*) or chatroom*).mp. |
| 15 | (Fitness Tracker* or activity tracker*).mp. |
| 16 | Online Social Networks/ |
| 17 | Information Technology/ or medical informatics.mp. |
| 18 | Computer Assisted Therapy/ |
| 19 | Human Computer Interaction/ or human computer interaction*.mp. |
| 20 | or/1-19 |
| 21 | Engag*.mp. |
| 22 | (attrition or patient dropouts).mp. |
| 23 | adher*.mp. |
| 24 | TREATMENT COMPLIANCE/ or COMPLIANCE/ |
| 25 | Patient Participation.mp. or Client Participation/ |
| 26 | ((intervention or online or web*) adj3 (adopt* or uptake or retention or maintenance or efficacy or us* or reach* or participat* or exposure)).mp. |
| 27 | (process adj (metric* or evaluation)).mp. |
| 28 | login*.mp. |
| 29 | (page* adj3 view*).mp. |
| 30 | ((module* or session*) adj complet*).mp. |
| 31 | (visit* adj3 website*).mp. |
| 32 | implement*.mp. |
| 33 | feasibil*.mp. |
| 34 | time spent online.mp. |
| 35 | (user adj3 (engag* or experience* or enjoyment or attention or interest or affect or immersion or involvement)).mp. |
| 36 | Subjective experience*.mp. |
| 37 | usability.mp. |
| 38 | or/21-37 |
| 39 | exp exercise/ |
| 40 | physical activit*.mp. |
| 41 | physical inactivit*.mp. |
| 42 | (physical education and training).mp. |
| 43 | Physical Fitness/ |
| 44 | exp SEDENTARY BEHAVIOR/ or sedentary.mp. |
| 45 | leisure time/ |
| 46 | exp sports/ |
| 47 | (exercise* adj aerobic*).mp. |
| 48 | sport*.mp. |
| 49 | ((lifestyle* or life style*) adj5 activ*).mp. |
| 50 | Motor Activity.mp. |
| 51 | or/39-50 |
| 52 | diets/ |
| 53 | healthy eating.mp. |
| 54 | fruit*.mp. |
| 55 | vegetable*.mp. |
| 56 | (canteen* or cafeteria*).mp. |
| 57 | food service*.mp. |
| 58 | calorie*.mp. |
| 59 | exp Food Intake/ or Energy Intake.mp. |
| 60 | energy density.mp. |
| 61 | Eating Behavior/ or feeding behavio*.mp. |
| 62 | dietary intake.mp. |
| 63 | food habits.mp. |
| 64 | FOOD/ |
| 65 | Dietary Fats.mp. |
| 66 | ((feeding or food or nutrition*) adj program*).mp. |
| 67 | nutrition/ or nutrition*.mp. |
| 68 | mealtimes/ or (meals or breakfast or lunch or snacks).mp. |
| 69 | menu*.mp. |
| 70 | (food adj purchas*).mp. |
| 71 | ((sweetened or sugar-sweetened or carbonated or soft) adj drink*).mp. |
| 72 | soda.mp. |
| 73 | or/52-72 |
| 74 | SMOKING CESSATION/ or exp TOBACCO SMOKING/ |
| 75 | smok*.mp. |
| 76 | NICOTINE/ |
| 77 | ((ceas* or cess* or prevent* or stop* or quit* or abstin* or abstain* or reduc*) adj5 (tobacco or nicotine)).mp. |
| 78 | or/74-77 |
| 79 | Health Promotion/ or Health Education/ or Health Behavior/ or Health Knowledge/ or health behav*.mp. |
| 80 | Lifestyle/ |
| 81 | Primary Prevention.mp. |
| 82 | Secondary Prevention.mp. |
| 83 | prevent* care.mp. |
| 84 | or/79-83 |
| 85 | exp Obesity/ or obes*.mp. |
| 86 | Weight Gain/ |
| 87 | Weight Loss/ |
| 88 | Body weight/ or weight manag*.mp. |
| 89 | weight control.mp. |
| 90 | Overweight/ or (over weight or overeat* or over eat*).mp. |
| 91 | weight change*.mp. |
| 92 | weight status.mp. |
| 93 | Body Mass Index/ or bmi.mp. |
| 94 | or/85-93 |
| 95 | exp Binge Drinking/ or exp Alcohol Drinking Patterns/ |
| 96 | alcohol*.mp. |
| 97 | alcoholism/ or alcohol intoxication/ |
| 98 | drink*.mp. |
| 99 | drunk.mp. |
| 100 | intoxicat*.mp. |
| 101 | or/95-100 |
| 102 | (prompt* or reminder* or (push adj notification*)).mp. |
| 103 | ((chat adj room*) or chatroom*).mp. |
| 104 | ((bulletin adj board*) or (message adj board*)).mp. |
| 105 | ((online adj social adj network*) or (social adj technolog*) or (social adj network* adj site*)).mp. |
| 106 | (tailor* or personali* or relevan* or individuali*).mp. |
| 107 | feedback.mp. |
| 108 | (Gamification or (gam* adj strateg*)).mp. |
| 109 | (Incentive* or reinforcement* or reward*).mp. |
| 110 | (goal adj setting).mp. |
| 111 | monitor*.mp. |
| 112 | ((leader adj board) or leaderboard or (progress adj report) or (progress adj chart)).mp. |
| 113 | (multimedia messag* or MMS).mp. |
| 114 | (text message* or short messag* service* or SMS).mp. |
| 115 | or/102-114 |
| 116 | Clinical Trials/ |
| 117 | PLACEBO/ |
| 118 | Experimental Design/ |
| 119 | (random* or trial* or placebo* or control* or comparison group* or single blind or double blind or quasiexperimental or quasi experimental or pseudo experimental).mp. |
| 120 | or/116-119 |
| 121 | or/39-100 |
| 122 | 20 and 38 and 115 and 120 and 121 |
| **123** | **limit 122 to (human and english language)** |

**CINAHL**

| **#** | **Query** |
| --- | --- |
| S1 | phone n1 app* |
| S2 | (MH "Cellular Phone") OR (MH "Smartphone") OR (MH "Telephone") |
| S3 | (MH "Mobile Applications") |
| S4 | ("mobile device*" or smartphone*) |
| S5 | (MH "Virtual Reality") |
| S6 | (MH "Online Systems") OR "online" |
| S7 | (MH "Internet") OR (MH "Social Media") OR (MH "World Wide Web") |
| S8 | ((internet or web) n1 based) |
| S9 | ("world wide web" or WWW or website*) |
| S10 | (MH "Electronic Mail") OR "e-mail*" OR email* |
| S11 | ((mobile or cellular or cell or smart) n1 (phone* or telephone*)) |
| S12 | (MH "Telemedicine") OR e-health or ehealth or "electronic health" |
| S13 | "m-health" or mhealth or "mobile health" |
| S14 | (("digital health" or digital) n1 intervention*) |
| S15 | (interactive n1 ((health n1 communicat*) or video* or technolog* or multimedia)) |
| S16 | ((chat n1 room*) or chatroom*) |
| S17 | (MH "Fitness Trackers") OR "activity tracker*" |
| S18 | (MH "Computer Communication Networks") |
| S19 | (MH "Medical Informatics") |
| S20 | (MH "Information Technology") |
| S21 | (MH "Therapy, Computer Assisted") |
| S22 | (MH "User-Computer Interface") OR "human computer interaction*" |
| S23 | S1 OR S2 OR S3 OR S4 OR S5 OR S6 OR S7 OR S8 OR S9 OR S10 OR S11 OR S12 OR S13 OR S14 OR S15 OR S16 OR S17 OR S18 OR S19 OR S20 OR S21 OR S22 |
| S24 | "Engag*" |
| S25 | "attrition" |
| S26 | (MH "Patient Dropouts") |
| S27 | adher* |
| S28 | (MH "Patient Compliance") OR "COMPLIANCE" |
| S29 | (MH "Consumer Participation") OR "Patient Participation" |
| S30 | ((intervention or online or web*) n3 (adopt* or uptake or retention or maintenance or efficacy or us* or reach* or participat* or exposure)) |
| S31 | (process n1 (metric* or evaluation)) |
| S32 | login* |
| S33 | (page* n3 view*) |
| S34 | ((module* or session*) n1 complet*) |
| S35 | (visit* n3 website*) |
| S36 | implement* |
| S37 | feasibil* |
| S38 | "time spent online" |
| S39 | (user n3 (engag* or experience* or enjoyment or attention or interest or affect or immersion or involvement)) |
| S40 | "Subjective experience*" |
| S41 | usability |
| S42 | S24 OR S25 OR S26 OR S27 OR S28 OR S29 OR S30 OR S31 OR S32 OR S33 OR S34 OR S35 OR S36 OR S37 OR S38 OR S39 OR S40 OR S41 |
| S43 | (MH "Exercise+") |
| S44 | (MH "Physical Activity") OR "physical activit*" |
| S45 | "physical inactivit*" |
| S46 | (MH "Physical Education and Training") OR ""physical education and training"" |
| S47 | (MH "Physical Fitness") |
| S48 | sedentary |
| S49 | (MH "Leisure Activities+") |
| S50 | (MH "Sports+") |
| S51 | (exercise* n1 aerobic*) |
| S52 | sport* |
| S53 | ((lifestyle* or life style*) n5 activ*) |
| S54 | (MH "Motor Activity") |
| S55 | (MH "Diet+") |
| S56 | "healthy eating" |
| S57 | (MH "Fruit") OR "Fruit*" |
| S58 | (MH "Vegetables") OR "vegetable*" |
| S59 | canteen* or cafeteria* |
| S60 | (MH "Food Services") OR "food service*" |
| S61 | calorie* |
| S62 | (MH "Energy Intake") |
| S63 | (MH "Energy Density") |
| S64 | (MH "Eating Behavior") OR "feeding behavio*" |
| S65 | (MH "Eating") |
| S66 | "dietary intake" OR (MH "Food Intake") |
| S67 | (MH "Food Habits") |
| S68 | (MH "Food") |
| S69 | (MH "Dietary Fats") OR (MH "Fats, Unsaturated") |
| S70 | ((feeding or food or nutrition*) n1 program*) |
| S71 | (MH "Nutritional Status") |
| S72 | (MH "Child Nutrition Disorders") OR (MH "Total Parenteral Nutrition") OR "nutrition*" |
| S73 | (MH "Meals") OR (MH "Breakfast") OR (MH "Lunch") OR (MH "Snacks") |
| S74 | (MH "Menu Planning") OR "menu*" |
| S75 | (food n1 purchas*) |
| S76 | (MH "Carbonated Beverages") OR (soft drink or soda) |
| S77 | ((sweetened or sugar-sweetened) n1 drink*) |
| S78 | (MH "Smoking") OR (MH "Smoking Cessation") |
| S79 | smok* |
| S80 | (MH "Nicotine") |
| S81 | (MH "Tobacco") |
| S82 | ((ceas* or cess* or prevent* or stop* or quit* or abstin* or abstain* or reduc*) n5 (tobacco or nicotine)) |
| S83 | (MH "Health Promotion") |
| S84 | (MH "Health Education") |
| S85 | (MH "Health Behavior") OR "health behav*" |
| S86 | (MH "Health Knowledge") |
| S87 | (MH "Life Style") |
| S88 | "Primary Prevention" |
| S89 | "Secondary Prevention" |
| S90 | (MH "Preventive Health Care") OR "prevent* care" |
| S91 | (MH "Obesity") OR (MH "Pediatric Obesity") OR (MH "Obesity, Morbid") OR "obes*" |
| S92 | (MH "Weight Gain") |
| S93 | (MH "Weight Loss") |
| S94 | (MH "Body Weight") OR "weight manag*" |
| S95 | (MH "Weight Control") |
| S96 | (overweight or "over weight" or overeat* or "over eat*") |
| S97 | (MH "Body Weight Changes") OR "weight change*" |
| S98 | "weight status" |
| S99 | "bmi" |
| S100 | (MH "Alcohol Drinking") OR (MH "Binge Drinking") |
| S101 | alcohol* |
| S102 | (MH "Alcoholism") OR (MH "Alcoholic Intoxication") |
| S103 | drink* |
| S104 | drunk |
| S105 | intoxicat* |
| S106 | S43 OR S44 OR S45 OR S46 OR S47 OR S48 OR S49 OR S50 OR S51 OR S52 OR S53 OR S54 OR S55 OR S56 OR S57 OR S58 OR S59 OR S60 OR S61 OR S62 OR S63 OR S64 OR S65 OR S66 OR S67 OR S68 OR S69 OR S70 OR S71 OR S72 OR S73 OR S74 OR S75 OR S76 OR S77 OR S78 OR S79 OR S80 OR S81 OR S82 OR S83 OR S84 OR S85 OR S86 OR S87 OR S88 OR S89 OR S90 OR S91 OR S92 OR S93 OR S94 OR S95 OR S96 OR S97 OR S98 OR S99 OR S100 OR S101 OR S102 OR S103 OR S104 OR S105 |
| S107 | (prompt* or reminder* or (push n1 notification*)) |
| S108 | ((chat n1 room*) or chatroom*) |
| S109 | ((bulletin n1 board*) or (message n1 board*)) |
| S110 | ((online n1 social n1 network*) or (social n1 technolog*) or (social n1 network* n1 site*)) |
| S111 | (tailor* or personali* or relevan* or individuali*) |
| S112 | feedback |
| S113 | (Gamification or (gam* n1 strateg*)) |
| S114 | (Incentive* or reinforcement* or reward*) |
| S115 | (goal n1 setting) |
| S116 | monitor* |
| S117 | ((leader n1 board) or leaderboard or (progress n1 report) or (progress n1 chart)) |
| S118 | ("multimedia messag*" or MMS) |
| S119 | ("text message*" or "short messag* service*" or SMS) |
| S120 | S107 OR S108 OR S109 OR S110 OR S111 OR S112 OR S113 OR S114 OR S115 OR S116 OR S117 OR S118 OR S119 |
| S121 | (MH "Clinical Trials") |
| S122 | (MH "Random Assignment") OR "random allocation" |
| S123 | (MH "Double-Blind Studies") |
| S124 | (MH "Single-Blind Studies") |
| S125 | (MH "Placebos") |
| S126 | (MH "Study Design") OR "Research Design" |
| S127 | (MH "Evaluation Research") OR "Evaluation Studies" |
| S128 | (MH "Comparative Studies") |
| S129 | "Cross-Over Studies" OR (MH "Crossover Design") |
| S130 | TI placebo* OR AB placebo* |
| S131 | TI random* OR AB random* |
| S132 | TI control* OR AB control* |
| S133 | TI "comparison group*" OR AB "comparison group*" |
| S134 | TI ( (quasiexperimental or "quasi experimental" or "pseudo experimental") ) OR AB ( (quasiexperimental or "quasi experimental" or "pseudo experimental") ) |
| S135 | S121 OR S122 OR S123 OR S124 OR S125 OR S126 OR S127 OR S128 OR S129 OR S130 OR S131 OR S132 OR S133 OR S134 |
| **S136** | **S23 AND S42 AND S106 AND S120 AND S135** |

**CENTRAL – search in Title, Abstract and Keywords**

((mobile or cellular or cell or smart or app*) and (phone* or telephone*)) or “mobile application*” or “mobile device” or “virtual reality” or online or internet or blogging or “social media” or “world wide web” or www or website* or “e-mail” or email or “electronic mail” or telemedicine or “e-health” or ehealth or “electronic health” or “m-health” or “mobile health” or mhealth or “digital health” or “digital intervention” or “interactive health communication” or video or technolog* or multimedia or chatroom* or (chat and room*) or “activity tracker*” or “fitness tracker*” or “computer communication network*” or “medical informatics” or “computer assisted therapy” or “user computer interface*” or “human computer interaction”

Engag* or attrition or “patient dropout*” or adher* or compliance or “patient participation” or ((intervention or online or web*) near/3 (adopt* or uptake or retention or maintenance or efficacy or us* or reach* or participat* or exposure)) or (process near/1 (metric* or evaluation)) or login* or (page near/3 view*) or ((module* or session*) near/1 complet*) or (visit* near/3 website*) or implement* or feasibil* or “time spent online” or (user near/3 (engag* or experience* or enjoyment or attention or interest or affect or immersion or involvement)) or “subjective experience*” or usability

Exercise or “physical activit*” or “physical inactivit*” or “physical education and training” or “physical fitness” or “leisure activit*” or sport* or ((lifestyle* or life style*) near/5 activ*) or “motor activity” or diet or “healthy eating” or fruit* or vegetable* or canteen* or cafeteria* or “food service*” or calorie* or “energy intake” or “energy density” or “feeding behavio*” or eating or “dietary intake” or food or “dietary fats” or ((feeding or food or nutrition*) near/1 program*) or nutrition* or meals or breakfast or dinner or lunch or snack* or menu* or “food purchas*” or “carbonated beverage*” or “soft drink*” or soda or “sweetened drink*” or smok* or tobacco or nicotine or “health promotion” or “health education” or “health behavio*” or “health knowledge” or “life style” or lifestyle or “primary prevention” or “secondary prevention” or “prevent* care” or obes* or “weight gain” or “weight loss” or “body weight” or “weight manag*” or “weight control” or overweight or “over weight” or overeat* or “over eat*” or “weight change*” or “weight status” or “body mass index” or bmi or alcohol* or drink* or drunk* or intoxicat*

Prompt* or reminder* or “push notification*” or “chat room*” or chatroom* or “bulletin board*” or “message board*” or “social network” or “social technology” or tailor* or personali* or relevan* or individuali* or feedback or gamification or (gam* near/1 strategy*) or incentive* or reinforcement* or reward* or “goal setting” or monitor* or “leader board*” or leaderboard* or “progress report*” or “progress chart*” or “multimedia message*” or mms or “text message*” or “short messag* service*” or sms

Random* or “clinical trial” or “double blind” or “single blind” or placebo* or “research design” or “evaluation stud*” or “comparative stud*” or “cross over stud*” or control* or “comparison group*” or quasiexperimental or “quasi experimental” or “pseudo experimental”

**Scopus**

## TITLE-ABS ( ( ( mobile  OR  cellular  OR  cell  OR  smart  OR  app* )  W/1  ( phone*  OR  telephone* ) )  OR  "mobile application*"  OR  "mobile device"  OR  "virtual reality"  OR  online  OR  internet  OR  blogging  OR  "social media"  OR  "world wide web"  OR  www  OR  website*  OR  "e-mail"  OR  email  OR  "electronic mail"  OR  telemedicine  OR  "e-health"  OR  ehealth  OR  "electronic health"  OR  "m-health"  OR  "mobile health"  OR  mhealth  OR  "digital health"  OR  "digital intervention"  OR  "interactive health communication"  OR  video  OR  technolog*  OR  multimedia  OR  chatroom*  OR  ( chat  W/1  room* )  OR  "activity tracker*"  OR  "fitness tracker*"  OR  "computer communication network*"  OR  "medical informatics"  OR  "computer assisted therapy"  OR  "user computer interface*"  OR  "human computer interaction" )  AND  TITLE-ABS ( engag*  OR  attrition  OR  "patient dropout*"  OR  adher*  OR  compliance  OR  "patient participation"  OR  ( ( intervention  OR  online  OR  web* )  W/3  ( adopt*  OR  uptake  OR  retention  OR  maintenance  OR  efficacy  OR  us*  OR  reach*  OR  participat*  OR  exposure ) )  OR  ( process  W/1  ( metric*  OR  evaluation ) )  OR  login*  OR  ( page  W/3  view* )  OR  ( ( module*  OR  session* )  W/1  complet* )  OR  ( visit*  W/3  website* )  OR  implement*  OR  feasibil*  OR  "time spent online"  OR  ( user  W/3  ( engag*  OR  experience*  OR  enjoyment  OR  attention  OR  interest  OR  affect  OR  immersion  OR  involvement ) )  OR  "subjective experience*"  OR  usability )  AND  TITLE-ABS ( exercise  OR  "physical activit*"  OR  "physical inactivit*"  OR  "physical education and training"  OR  "physical fitness"  OR  "leisure activit*"  OR  sport*  OR  ( ( lifestyle*  OR  life  AND style* )  AND  activ* )  OR  "motor activity"  OR  diet  OR  "healthy eating"  OR  fruit*  OR  vegetable*  OR  canteen*  OR  cafeteria*  OR  "food service*"  OR  calorie*  OR  "energy intake"  OR  "energy density"  OR  "feeding behavio*"  OR  eating  OR  "dietary intake"  OR  food  OR  "dietary fats"  OR  ( ( feeding  OR  food  OR  nutrition* )  AND  program* )  OR  nutrition*  OR  meals  OR  breakfast  OR  dinner  OR  lunch  OR  snack*  OR  menu*  OR  "food purchas*"  OR  "carbonated beverage*"  OR  "soft drink*"  OR  soda  OR  "sweetened drink*"  OR  smok*  OR  tobacco  OR  nicotine  OR  "health promotion"  OR  "health education"  OR  "health behavio*"  OR  "health knowledge"  OR  "life style"  OR  lifestyle  OR  "primary prevention"  OR  "secondary prevention"  OR  "prevent* care"  OR  obes*  OR  "weight gain"  OR  "weight loss"  OR  "body weight"  OR  "weight manag*"  OR  "weight control"  OR  overweight  OR  "over weight"  OR  overeat*  OR  "over eat*"  OR  "weight change*"  OR  "weight status"  OR  "body mass index"  OR  bmi  OR  alcohol*  OR  drink*  OR  drunk*  OR  intoxicat* )  AND  TITLE-ABS ( prompt*  OR  reminder*  OR  "push notification*"  OR  "chat room*"  OR  chatroom*  OR  "bulletin board*"  OR  "message board*"  OR  "social network"  OR  "social technology"  OR  tailor*  OR  personali*  OR  relevan*  OR  individuali*  OR  feedback  OR  gamification  OR  ( gam*  W/1  strategy* )  OR  incentive*  OR  reinforcement*  OR  reward*  OR  "goal setting"  OR  monitor*  OR  "leader board*"  OR  leaderboard*  OR  "progress report*"  OR  "progress chart*"  OR  "multimedia message*"  OR  mms  OR  "text message*"  OR  "short messag* service*"  OR  sms )  AND  TITLE-ABS ( random*  OR  "clinical trial"  OR  "double blind"  OR  "single blind"  OR  placebo*  OR  "research design"  OR  "evaluation stud*"  OR  "comparative stud*"  OR  "cross over stud*"  OR  control*  OR  "comparison group*"  OR  quasiexperimental  OR  "quasi experimental"  OR  "pseudo experimental" )  AND  ( LIMIT-TO ( EXACTKEYWORD ,  "Human" )  OR  LIMIT-TO ( EXACTKEYWORD ,  "Humans" ) )  AND  ( LIMIT-TO ( LANGUAGE ,  "English" ) )

**Academic Search Ultimate – search in Title and Abstract**

((mobile or cellular or cell or smart or app*) n1 (phone* or telephone*)) or “mobile application*” or “mobile device” or “virtual reality” or online or internet or blogging or “social media” or “world wide web” or www or website* or “e-mail” or email or “electronic mail” or telemedicine or “e-health” or ehealth or “electronic health” or “m-health” or “mobile health” or mhealth or “digital health” or “digital intervention” or “interactive health communication” or video or technolog* or multimedia or chatroom* or (chat n1 room*) or “activity tracker*” or “fitness tracker*” or “computer communication network*” or “medical informatics” or “computer assisted therapy” or “user computer interface*” or “human computer interaction”

Engag* or attrition or “patient dropout*” or adher* or compliance or “patient participation” or ((intervention or online or web*) n3 (adopt* or uptake or retention or maintenance or efficacy or us* or reach* or participat* or exposure)) or (process n1 (metric* or evaluation)) or login* or (page n3 view*) or ((module* or session*) n1 complet*) or (visit* n3 website*) or implement* or feasibil* or “time spent online” or (user n3 (engag* or experience* or enjoyment or attention or interest or affect or immersion or involvement)) or “subjective experience*” or usability

Exercise or “physical activit*” or “physical inactivit*” or “physical education and training” or “physical fitness” or “leisure activit*” or sport* or ((lifestyle* or life style*) n5 activ*) or “motor activity” or diet or “healthy eating” or fruit* or vegetable* or canteen* or cafeteria* or “food service*” or calorie* or “energy intake” or “energy density” or “feeding behavio*” or eating or “dietary intake” or food or “dietary fats” or ((feeding or food or nutrition*) n1 program*) or nutrition* or meals or breakfast or dinner or lunch or snack* or menu* or “food purchas*” or “carbonated beverage*” or “soft drink*” or soda or “sweetened drink*” or smok* or tobacco or nicotine or “health promotion” or “health education” or “health behavio*” or “health knowledge” or “life style” or lifestyle or “primary prevention” or “secondary prevention” or “prevent* care” or obes* or “weight gain” or “weight loss” or “body weight” or “weight manag*” or “weight control” or overweight or “over weight” or overeat* or “over eat*” or “weight change*” or “weight status” or “body mass index” or bmi or alcohol* or drink* or drunk* or intoxicat*

Prompt* or reminder* or “push notification*” or “chat room*” or chatroom* or “bulletin board*” or “message board*” or “social network” or “social technology” or tailor* or personali* or relevan* or individuali* or feedback or gamification or (gam* n1 strategy*) or incentive* or reinforcement* or reward* or “goal setting” or monitor* or “leader board*” or leaderboard* or “progress report*” or “progress chart*” or “multimedia message*” or mms or “text message*” or “short messag* service*” or sms

Random* or “clinical trial” or “double blind” or “single blind” or placebo* or “research design” or “evaluation stud*” or “comparative stud*” or “cross over stud*” or control* or “comparison group*” or quasiexperimental or “quasi experimental” or “pseudo experimental”
