# Supplementary material for: The Effectiveness of Strategies to Improve User Engagement With Digital Health Interventions Targeting Nutrition, Physical Activity, and Overweight and Obesity: Systematic Review and Meta-Analysis
Source: J Med Internet Res. 2023 Dec 19;25:e47987. doi: 10.2196/47987 (PMC10762625; doi:10.2196/47987)
Supplement: Multimedia Appendix 2 [file jmir_v25i1e47987_app2.docx]

**Multimedia Appendix 2. Engagement Strategy Codes and Examples of Coded Strategies**

| **Behavior Change Techniques (Michie 2011)** | **Example of coded strategies** |
| --- | --- |
| Goals and planning (GP) | Phone calls to set goals for physical activity, using motivational interviewing to set personal goals, automatically generated personal behavior targets |
| Feedback and monitoring (FM) | Automated feedback on performance, self-monitoring of performance |
| Social support (SS) | Web-based coach to provide support and encouragement, features allowing interaction with peers (eg, web-based forums, virtual walking groups, and group discussions) |
| Shaping knowledge (SK) | Physical activity training sessions, educational audio files, lessons on how to perform nutrition–related behavior change (eg, reducing sugary foods, reading food labels, shopping tips) |
| Natural consequences (NC) | Weekly emails with information on consequences of behavior |
| Comparison of behavior (CB) | Demonstration of the behavior (eg, via cooking videos, nutrition-based competitions between peers via Facebook) |
| Associations (As) | Weekly emails prompting participants to use the DHI, use of regular text messages to prompt and motivate health behavior change. |
| Repetition and substitution (RS) | Discussion of physical activity goals and principles of habit formation with a trained staff member; weekly skills training emails prompting generalization of a target behavior |
| Comparison of outcomes (CO) | Comparison of outcomes via credible source, comparative imaging of future outcomes  Exploration/discussion of the pros and cons of weight loss during a group discussion |
| Reward and threat (RT) | Financial incentives, comparing the chance to receive rewards or losing rewards, virtual points to receive badges, medals or to redeem for gifts |
| Regulation (R) | Entry of positive and negative moods into a monitoring interface, receipt of coping strategies, and development of coping skills |
| Antecedents (AN) | Tips to avoid or distract from unwanted behaviors, the use of environmental cues to promote healthy habits |
| Identity (I) | Nutrition-related resources encouraging participants to re-evaluate beliefs |
| Scheduled consequences (SC) | Loss and regret aversion messaging, removal of rewards or incentives contingent upon targeted behavior and/or outcomes |
| Self-belief (SB) | Creation of audio recordings of positive events they were looking forward to and able to clearly imagine |
| Covert learning (CL) | Not coded |

| **Design features (Webb 2010)** | **Example applications** |
| --- | --- |
| *Automated functions* |  |
| (a) the use of an enriched information  environment (eg, supplementary content and links, testimonials, videos, or games) | Supplementary use of video to deliver tailored feedback, training to pre-record audio for “episodic thinking”, additional emails delivering BCTs, additional automated progress feedback reports, use of a mood- monitoring interface |
| (b) automated tailored feedback based on  individual progress monitoring (eg, comparison to norms or goals, reinforcing messages, or coping messages) | Automated weekly personalised feedback based on health behavior data entries into DHI, use of an automated computer assistant to monitor progress and provide feedback |
| (c) automated follow-up messages (eg, reminders, tips, newsletters, encouragement) | Notifications to encourage entry of data into DHI, daily support text messages, support messages to prompt health behavior targeted by DHI |
| *Communicative functions* |  |
| (d) access to an advisor to request advice (eg, “ask the expert” facility, expert-led discussion board, or chat sessions) | Discussion board with new topics posted weekly by the expert, who also responded to participant comments |
| (e) scheduled contact with advisor (eg, emails) | Biweekly video-coaching sessions in which participant physical activity feedback was discussed and encouragement and support provided; email counselling sessions using motivational interviewing to support dietary change; structured phone calls of gradually decreasing frequency covering behavioral weight loss techniques with interventionists |
| (f) peer-to-peer access (eg, buddy systems, peer-to-peer discussions boards, forums, or live chat) | Allowing access to other program participants’ Facebook pages for support with health behavior of interest, creation of friend lists, ability to post to group forums, and virtual walking groups |
| *Supplementary modes* |  |
| (g) email | Emails with weight and dietary feedback; emails summarising weekly physical activity information with link prompting access to associated website content |
| (h) telephone | Additional telephone coaching for weight management |
| (i) SMS text messages | Nutrition SMS text messages including a link prompting access to associated website content; SMS text messages prompting engagement in physical activity; SMS text messages prompting recording of vegetable intake |
| (j) CD-ROM | Not coded |
| (k) videoconferencing | Biweekly video-coaching sessions |
