# Supplementary material for: The Effectiveness of Strategies to Improve User Engagement With Digital Health Interventions Targeting Nutrition, Physical Activity, and Overweight and Obesity: Systematic Review and Meta-Analysis
Source: J Med Internet Res. 2023 Dec 19;25:e47987. doi: 10.2196/47987 (PMC10762625; doi:10.2196/47987)
Supplement: Multimedia Appendix 3 [file jmir_v25i1e47987_app3.docx]

**Multimedia Appendix 3. Characteristics of included studies**

| First author, year  (country) | Study design, DHI type, participants, sample size, type of engagement outcome | DHI intervention description and duration |
| --- | --- | --- |
|  |  |  |
| Alley,  2014 [60]  (Australia) | Design: *RCT*  DHI type: *Website*  Participants: *University staff and students aged >18 years*  Total N: *41*  Outcome: *Use, User experience* | Description: *A web-based physical activity program with or without video-tailored physical activity feedback*  Conditions:  *Arm 1: Website plus video-tailored feedback*  *Arm 2:* *Website plus text-tailored feedback*  Duration: *20–25 mins (one off session)* |
| Alley,  2016 [61]  (Australia) | Design: *RCT*  DHI type: *Website*  Participants: *Inactive adults*  Total N: *106*  Outcome: *Use, User experience* | Description: *A web-based physical activity program with computer-tailored advice with or without video-based coaching sessions*  Conditions:  *Arm 1: Website plus computer-tailored advice modules plus video coaching*  *Arm 2: Website plus computer-tailored advice modules*  Duration: *8 weeks* |
| Alley,  2023 [90, 115]  (Australia) | Design: *RCT*  DHI type: *Website*  Participants: *Adults aged ≥65 years*  Total N: *174*  Outcome: *Use, User experience* | Description: *A web-based physical activity intervention in older adults with or without Fitbits*  Conditions:  *Arm 1: Website with tailored advice plus activity tracker*  *Arm 2: Website with tailored advice*  Duration: *12 weeks* |
| Beleigoli,  2020 [91]  (Brazil) | Design: *RCT*  DHI type: *Website*  Participants: *University students and staff aged 18 to 60 years, with a BMI ≥25* *kg/m^2^*  Total N: *828*  Outcome: *Use* | Description: *Web-based platform for healthy weight loss with or without personalized coaching*  Conditions:  *Arm 1: Web-based platform plus personalized coaching*  *Arm 2: Web-based platform*  Duration: *24 weeks* |
| Blanson Henkemans, 2009 [62]  (Netherlands) | Design: *RCT*  DHI type: *Website*  Participants: *Overweight adults*  Total N: *191*  Outcome: *Use, User experience* | Description: *A web-based lifestyle diary with or without a computer assistant providing feedback on self-management*  Conditions:  *Arm 1: Website plus computer assistant providing feedback*  *Arm 2: Website only*  Duration: *4 weeks* |
| Brindal,  2012 [63]  (Australia) | Design: *RCT*  DHI type: *Website*  Participants: *Adults with a BMI >25 kg/m^2^*  Total N: *8112*  Outcome: *Use, User experience* | Description: *A web-based weight loss program with or without supportive features and personalization*  Conditions:  *Arm 1: Enhanced website plus personalized interactive meal planner*  *Arm 2: Enhanced website plus interactive meal planner*  *Arm 3: Standard Website*  Duration: *12 weeks* |
| Brindal,  2019 [64] (Australia) | Design: *RCT*  DHI type: *Mobile app*  Participants*: Adults aged ≥18 years, lost ≥5% of their body weight within the last 2 years*  Total N: *88*  Outcome: *Use, User experience* | Description: *An app-based weight loss program with or without persuasive and interactive features*  Conditions:  *Arm 1: Enhanced mobile app*  *Arm 2: Standard mobile app*  Duration: *12 weeks* |
| Butryn, 2020 [92]  (United States) | Design: *RCT*  DHI type: *Activity tracker, smart scale, mobile app*  Participants: *Adults aged 18 to 70 years with a BMI 25-45 kg/m^2^*  Total N: *77*  Outcome: *Use, User experience* | Description: *Digital self-monitoring of weight, physical activity and food intake, with or without counsellor support*  Conditions:  *Arm 1: Activity tracker plus smart scale plus mobile app plus counsellor support*  *Arm 2:* *Activity tracker plus smart scale plus mobile app*  Duration: *52 weeks* |
| Chai,  2019 [65, 116]  (Australia) | Design: *RCT*  DHI type: *Telehealth, website*  Participants: *Children aged 4 to 11 years with BMI ≥21.5 kg/m^2^ and their parent/s or legal guardian*  Total N: *31*  Outcome: *Use, User experience* | Description: *A web-based telehealth weight management program with or without evidence-based nutrition SMS text messages*  Conditions:  *Arm 1: Website plus telehealth plus SMS text messages*  *Arm 2:* *Website plus telehealth*  Duration: *12 weeks* |
| Couper,  2010 [66]  (United States) | Design: *RCT*  DHI type: *Website*  Participants: *Adults aged 21 to 65 years who had at least 1 year enrolment in the health care plan of participating health care systems*  Total N: *2513*  Outcome: *Use* | Description: *A web-based nutrition program with or without tailored content and email counselling support sessions*  Conditions:  *Arm 1: Tailored website plus email counselling support*  *Arm 2: Tailored website*  *Arm 3: Untailored website*  Duration: *12 months* |
| Dennison,  2014 [67]  (United Kingdom) | Design: *RCT*  DHI type: *Website*  Participants: *Adults with BMI of ≥23 kg/m^2^*  Total N: *511*  Outcome: *Use* | Description: *A web-based weight management program, with or without brief telephone support*  Conditions:  *Arm 1: Website plus telephone coaching*  *Arm 2: Website*  Duration: *12 weeks* |
| Edney,  2019 – 2020 [111, 117]  (Australia) | Design: *Cluster RCT*  DHI type: *Mobile app*  Participants: *Adults aged 18 to 65 years, who used Facebook weekly, reported as completing <150 minutes of MVPA per week*  Total N: *81 teams / 301 participants*  Outcome: *Use* | Description: *An app-based physical activity program with or without gamified and social components via Facebook*  Conditions:  *Arm 1: Gamified mobile app*  *Arm 2: Basic mobile app*  Duration: *100 days* |
| Eisenhauer, 2021 [93]  (United States) | Design: *Feasibility RCT*  DHI type: *Mobile app*  Participants: *Rural male adults, aged 40 to 69 years with a BMI ≥28 kg/m^2^ (but not weighing >396 pounds)*  Total N: *80*  Outcome: *Use* | Description: *An app-based weight loss program with or without personalized reports, goal-setting, and the opportunity to participate in a discussion board*  Conditions:  *Arm 1: Premium mobile app*  *Arm 2: Basic mobile app*  Duration: *12 weeks* |
| Ellingson,  2019 [68]  (United States) | Design: *RCT*  DHI type: *Activity tracker*  Participants: *Adults aged 24 to 65 years who were engaging in <150 minutes of moderate or <75 minutes of vigorous activity per week*  Total N: *91*  Outcome: *Use* | Description: *A wearable physical activity monitoring device (Fitbit) with or without motivational interviewing (MI) -based coaching and habit formation education*  Conditions:  *Arm 1: Activity tracker plus MI and habit education*  *Arm 2: Activity tracker*  Duration: *12 weeks* |
| Fanning,  2017 [105]  (United States) | Design: *Factorial RCT*  DHI type: *Mobile app*  Participants: *Healthy, low-active adults (not engaging in ≥30 minutes of MVPA on ≥2 days per week) aged 30 to 54 years*  Total N: *116*  Outcome: *Use* | Description*: An app-based physical activity program with or without feedback, incremental rewards and the opportunity to set proximal and distal goals*  Conditions:  *Arm 1: Mobile app plus goal setting module plus point-based feedback module*  *Arm 2: Mobile app plus goal setting module*  *Arm 3: Mobile app plus point-based feedback module*  *Arm 4: Mobile app*  Duration: *12 weeks* |
| Forman, 2019 [94]  (United States) | Design: *RCT*  DHI type: *Mobile app*  Participants: *Adults aged 18 to 70 with a BMI 25-50 kg/m^2^ who owned an iPhone with a data plan*  Total N: *181*  Outcome: *User experience* | Description: *An app-based dietary monitoring program with or without personalized feedback regarding triggers of dietary lapses*  Conditions:  *Arm 1: Self-monitoring plus personalized feedback*  *Arm 2: Self-monitoring*  Duration: *10 weeks* |
| Gabriele,  2009 - 2011 [69, 114]  (United States) | Design: *RCT*  DHI type: *Website*  Participants: *University employees aged 30 to 60 years with a BMI of 25–40 kg/m^2^*  Total N: *104*  Outcome: *Use* | Description: *A weight loss e-coaching program with or without directive and non-directive support*  Conditions:  *Arm 1: Website plus directive support*  *Arm 2: Website plus nondirective support*  *Arm 3: Website plus minimal support*  Duration: *12 weeks* |
| Granet, 2023 [95]  (Canada) | Design: *Feasibility RCT*  DHI type: *Telehealth*  Participants: *Adults aged 60 years and over who were inactive (<7500 steps per day and <150 minutes of exercise per week) and were not frail*  Total N: *46*  Outcome: *Use, User experience* | Description: *A web-based physical exercise intervention with varying frequencies of live vs. pre-recorded sessions*  Conditions:  *Arm 1: 2 live and 1 pre-recorded sessions per week*  *Arm 2:  1 live and 2 pre-recorded sessions per week*  Duration: *12 weeks* |
| Guagliano,  2019 [110]  (United Kingdom) | Design: *Feasibility cluster RCT*  DHI type: *Website*  Participants: *Children aged 8 to 10 years (+ at least 1 adult responsible for their care and living in their main household)*  Total N: *12 families (clusters) / 18 adults*  Outcome: *Use, User experience* | Description: *A web-based child-led family physical activity program with or without all involvement of all family members in wearing pedometers and working together towards collective goals*  Conditions:  *Arm 1: Website plus all family members wore pedometer*  *Arm 2: Website plus only the child wore the pedometer*  Duration: *6 weeks* |
| Haslam, 2023 [96]  (Australia) | Design: *RCT*  DHI type: *Website*  Participants: *Adults aged 18 to 24 years with a BMI ≥18·5 kg/m^2^*  Total N: *662*  Outcome: *User experience* | Description: *A nutrition-focused website and dietary feedback with and without video support from a dietitian*  Conditions:  *Arm 1: Website plus dietary feedback plus dietitian support*  *Arm 2: Website plus dietary feedback*  Duration: *12 months* |
| Hutchesson, 2016; [70]  Collins,  2013 [118];  Collins 2012 [119]  (Australia) | Design: *RCT*  DHI type: *Website*  Participants: *Overweight and obese adults with a BMI of 25-40 kg/m^2^, aged 18 to 60 years*  Total N: *301*  Outcome: *Use* | Description: *A web-based weight loss program with or without individualized feedback and reminders*  Conditions:  *Arm 1: Enhanced website plus personalized feedback and reminders*  *Arm 2:* *Basic website*  Duration: *12 weeks* |
| Jin,  2021 [97]  (United States) | Design: *RCT*  DHI type: *SMS text messages*  Participants: *Adults aged ≥18 years, having preference and ability to read and speak Spanish*  Total N: *59*  Outcome: *Use, User experience* | Description: *A physical activity and nutrition educational intervention delivered via SMS text messages with or without a phone call from a native Spanish-speaking health educator*  Conditions:  *Arm 1: SMS text messages + phone call*  *Arm 2: SMS text messages*  Duration: *12 weeks* |
| Kleimann,  2019 [71]  (United Kingdom) | Design: *RCT*  DHI type: *Mobile app*  Participants: *Adults aged ≥18 years with a BMI ≥25 kg/m^2^*  Total N: *55*  Outcome: *Use* | Description: *A habits-based weight loss app with or without additional self-regulation strategies*  Conditions:  *Arm 1: Mobile app plus self-regulation strategies*  *Arm 2: Standard mobile app*  Duration: *3 months* |
| Kolt,  2017 [103]  (Australia) | Design: *RCT*  DHI type: *Website*  Participants: *Sedentary adults who participate in <30 minutes of MVPA on ≥5 days per week*  Total N: *333*  Outcome: *Use, User experience* | Description: *A web-based physical activity program with or without social networking*  Conditions:  *Arm 1: Website plus social networking*  *Arm 2: Basic website*  Duration: *18 months* |
| Kwan,  2013 [108]  (Canada) | Design: *Cluster RCT*  DHI type: *Website*  Participants: *University students who lived on 1 of the 2 campus residence buildings participating in the study*  Total N: *4 university residence floors / 91 participants*  Outcome: *Use* | Description: *A web-based physical activity program targeting psychosocial mediating variables with or without weekly email prompts*  Conditions:  *Arm 1: Website plus email prompts*  *Arm 2: Website*  Duration: *6 weeks* |
| LaRose,  2019 [72]  (United States) | Design: *RCT*  DHI type: *Website*  Participants: *Adults aged 18 to 25 years with a BMI of ≥25 kg/m^2^*  Total N: *35*  Outcome: *Use, User experience* | Description: *A web-based behavioral weight loss program with or without competency-building group sessions*  Conditions:  *Arm 1: Web-based* *behavioral weight loss plus optional community-based competency-building group sessions (in person)*  *Arm 2: Web-based behavioral weight loss*  Duration: *3 months* |
| LaRose, 2020a  [98]  (United States) | Design: *RCT*  DHI type: *Website*  Participants: *Adults aged 18 to 25 years with a BMI of 25-45 kg/m^2^*  Total N: *47*  Outcome: *Use, User experience* | Description: *A web-based behavioral weight loss program with or without motivational interviewing*  Conditions:  *Arm 1: Web-based* *behavioral weight loss plus motivational interviewing and e-coaching*  *Arm 2: Web-based behavioral weight loss*  Duration: *12 weeks* |
| LaRose, 2020b  [99], Leahey 2020 [120]  (United States) | Design: *RCT*  DHI type: *Website*  Participants: *Adults aged 18 to 70 years with a BMI of ≥25 kg/m^2^*  Total N: *180*  Outcome: *Use* | Description: *A web-based behavioral weight loss program with or without financial incentives*  Conditions:  *Arm 1: Web-based* *behavioral weight loss plus financial incentives*  *Arm 2: Web-based behavioral weight loss*  Duration: *12 weeks* |
| Levin, 2022  [100]  (United States) | Design: *RCT*  DHI type: *Mobile app*  Participants: *Adults aged 18 years or older who owned an iOS or Android smartphone*  Total N: *68*  Outcome: *Use, User experience* | Description: *A health behavior tracking app with or without an Acceptance and Commitment Therapy app*  Conditions:  *Arm 1: Health behavior tracking app plus Acceptance and Commitment Therapy app*  *Arm 2: Health behavior tracking app*  Duration: *4 weeks* |
| Liao,  2020 [109]  (China) | Design: *Cluster RCT*  DHI type: *Activity tracker*  Participants: *Square dancers (from dancing groups) aged ≥45 years who practiced in square dancing at least once per week in the past 12 months*  Total N: *13 groups / 149 participants*  Outcome: *Use, User experience* | Description: *A wearable physical activity tracker with or without physical activity education, allocation to teams, and a dance training session*  Conditions:  *Arm 1: Activity tracker plus information-motivation-behavioral skills*  *Arm 2: Activity tracker*  Duration: *3 months* |
| Mailey,  2016 [73]  (United States) | Design: *RCT*  DHI type: *Website*  Participants: *Working mothers engaging in <150 minutes per week of MVPA*  Total N: *69*  Outcome: *Use, User experience* | Description: *A web-based physical activity and self-worth program with or without tasks to enhance group cohesion*  Conditions:  *Arm 1: Website plus enhanced discussion group*  *Arm 2: Website plus standard discussion group*  Duration: *8 weeks* |
| Micco,  2007 [74]  (United States) | Design: *RCT*  DHI type: *Website*  Participants: *Adults aged ≥18 years with a BMI of 25-39.9 kg/m^2^*  Total N: *123*  Outcome: *Use* | Description: *A web-based weight loss program with or without monthly in-person meetings*  Conditions:  *Arm 1: Website plus in-person support*  *Arm 2: Website*  Duration: *12 months* |
| Monroe,  2019 [75]  (United States) | Design: *RCT*  DHI type: *Website*  Participants*: Adults aged ≥18 years, with a BMI of 25-55 kg/m^2^, were in general good health, did not report losing ≥10% of initial body weight within the previous 6 months*  Total N: *36*  Outcome: *Use, User experience* | Description: *A web-based weight loss program with or without the provision of digital scales and fitness trackers to participant’s social networks*  Conditions:  *Arm 1: Evidence based behavioral weight loss program with website plus enhanced social climate (other adults to serve as support partners)*  *Arm 2: Evidence-based behavioral weight loss program with website*  Duration: *16 weeks* |
| Napolitano, 2013 [76]  (United States) | Design: *RCT*  DHI type: *Website*  Participants: *Healthy overweight and obese university students aged 18 to 29 years*  Total N: *35*  Outcome: *Use* | Description: *A weight loss program accessed via Facebook with or without daily SMS text messages, goal setting, self-monitoring, and social support strategies*  Conditions:  *Arm 1: Enhanced Facebook*  *Arm 2: Standard Facebook*  Duration: *8 weeks* |
| Newton Jr,  2014 [77]  (United States) | Design: *RCT*  DHI type: *Website*  Participants: *Sedentary children aged 6 to 10 years + a parent or legal guardian of each participating child.*  Total N: *27*  Outcome: *Use* | Description: *A web-based physical activity program with or without behavioral articles and SMS text message prompts*  Conditions:  *Arm 1: Enhanced website*  *Arm 2: Standard website*  Duration: *12 weeks* |
| Nour,  2019 [106]  (Australia) | Design: *Factorial RCT*  DHI type: *Mobile app*  Participants: *Adults aged 18 to 30 years*  Total N: *97*  Outcome: *Use* | Description: *An app-based nutrition program with or without a social support Facebook group and gamified features*  Conditions:  *Arm 1: Gamified mobile app plus Facebook*  *Arm 2: Standard mobile app plus Facebook*  *Arm 3: Gamified mobile app*  *Arm 4: Standard mobile app*  Duration: *4 weeks* |
| Nuijten,  2019 [107]  (Netherlands) | Design: *Cluster RCT*  DHI Type: *Mobile app*  Participants: *Staff and students from Eindhoven University of Technology*  Total N: *144*  Outcome: *Use* | Description: *An app-based healthy lifestyle virtual competition with or without 2 differently framed tangible rewards*  Conditions:  *Arm 1: Mobile app plus rewards framed as gains*  *Arm 2: Mobile app plus rewards framed as a loss*  *Arm 3: Mobile app (virtual point system only)*  Duration: *30 days* |
| Omran,  2018 [78]  (Canada) | Design: *RCT*  DHI type: *Website*  Participants: *Office-based university employees who were engaging in <150 minutes of MVPA per week or taking <10,000 steps per day*  Total N: *69*  Outcome: *Use* | Description: *A web-based physical activity program with or without financial incentives*  Conditions:  *Arm 1: Website plus incentive*  *Arm 2: Website*  Duration: *11 weeks* |
| Patel,  2019 [79]  (United States) | Design: *RCT*  DHI type: *Mobile app*  Participants: *Adults aged 21 to 65 years with a BMI of 25-45 kg/m^2^*  Total N: *105*  Outcome: *Use* | Description: *An app-based weight loss program with different self-monitoring strategies*  Conditions:  *Arm 1: Mobile app plus simultaneous tracking of body weight and nutrition*  *Arm 2: Mobile app plus tracking of body weight for 5 weeks prior to tracking nutrition*  *Arm 3: Mobile app plus tracking of nutrition*  Duration: *12 weeks* |
| Pischke, 2022  [101]  (Germany) | Design: *RCT*  DHI type: *Website, Mobile app*  Participants: *Adults aged 60 years or older who lived independently*  Total N: *129*  Outcome: *Use, User experience* | Description: *Self-monitoring of physical activity through use of a website, app, and optional face-to-face group sessions, with or without a physical activity tracker (Fitbit)*  Conditions:  *Arm 1: Website or app plus activity tracker*  *Arm 2: Website or app*  Duration: *10 weeks* |
| Pullen,  2008 [104]  (United States) | Design: *RCT*  DHI type: *Website*  Participants: *Rural women aged 50 to 69 years with a BMI of 25-34.9 kg/m^2^*  Total N: *21*  Outcome: *Use* | Description: *A web-based weight loss program with or without a web-based peer support group*  Conditions:  *Arm 1: Website plus support group*  *Arm 2: Website*  Duration: *3 months* |
| Ross,  2016 [80]  (United States) | Design: *RCT*  DHI type: *Activity tracker, Smart scale, Mobile app or Website.*  Participants: *Adults aged 18 to 70 years, with a BMI of 27-40 kg/m^2^*  Total N: *54*  Outcome: *Use* | Description: *Use of technology-based monitoring tools for weight loss with or without additional phone support*  Conditions:  *Arm 1: Mobile app/website plus activity tracker plus smart scale plus phone calls*  *Arm 2: Mobile app/website plus activity tracker plus smart scale*  Duration: *6 months* |
| Schoeppe, 2022 [102], Vandelanotte 2021 [112]  (Australia) | Design: *RCT*  DHI type: *Website*  Participants: *Adults aged 18 years or older with a BMI >17.5 kg/m^2^*  Total N: *334*  Outcome: *Use, User experience* | Description: *A web-based, personally tailored physical activity intervention with or without feedback delivered via video or text*  Conditions:  *Arm 1: Website plus video-tailored feedback*  *Arm 2:  Website plus text-tailored feedback*  *Arm 3: Website*  Duration: *3 months* |
| Shaw,  2012 [81]  (United States) | Design: *RCT*  DHI type: *SMS text messages*  Participants: *Adults ≥18 years who received treatment at the Duke Diet and Fitness Center (DFC) for weight loss, had a clinically measured BMI >30 kg/m^2^ at the start of DFC treatment, had completed a comprehensive diet and fitness program, lost 5% of their body weight*  Total N: *81*  Outcome: *Use, User experience* | Description: *A SMS text message program to sustain recent weight loss with promotion or prevention framed messages*  Conditions:  *Arm 1: Prevention-framed SMS text messages*  *Arm 2: Promotion-framed SMS text messages*  Duration: *1 month* |
| Soetens,  2014 [82]  Vandelanotte, 2011 [121]  (Australia) | Design: *RCT*  DHI type: *Website*  Participants: *Adults aged >18 years*  Total N: *863*  Outcome: *Use, User experience* | Description: *A web-based physical activity program with computer-tailored feedback provided in video and/or text format*  Conditions:  *Arm 1: Website plus video plus text-based computer feedback*  *Arm 2: Website plus video-based computer feedback*  *Arm 3: Website plus text-based computer feedback*  Duration: *One off session* |
| Sze,  2015 [83]  (United States) | Design: *RCT*  DHI type: *Website*  Participants: *Families with an overweight parent and an overweight child aged 8 to 12 years*  Total N: *20*  Outcome: *Use, User experience* | Description: *A weight loss program for families with web-based and mobile delivery of “episodic future thinking” strategies*  Conditions:  *Arm 1: Nutritional information plus episodic future thinking training*  *Arm 2: Nutrition information thinking only*  Duration: *4 weeks* |
| Tsai,  2007 [84]  (United States) | Design: *Feasibility RCT*  DHI type: *Mobile app*  Participants: *Clinically overweight or obese university students with a BMI ≥25 kg/m^2^, aged ≥18 years*  Total N: *10*  Outcome: *Use, User experience* | Description: *An app for weight management self-monitoring with SMS text message prompts (with different frequency of delivery) to record data*  Conditions:  *Arm 1: Mobile app plus 3 daily SMS text message prompts*  *Arm 2: Mobile app plus 1 daily SMS text message prompt*  Duration: *1 month* |
| Vandelanotte, 2017 [85]  (Australia) | Design: *Randomized ecological trial*  DHI type: *Website*  Participants: *Healthy adults aged ≥18 years*  Total N: *1328*  Outcome: *Use, User experience* | Description: *A web-based physical activity program with or without social networking capabilities*  Conditions:  *Arm 1: Enhanced website*  *Arm 2: Standard website*  Duration: *Unclear. Engagement measures are taken at 12 weeks* |
| Vandelanotte, 2018 [86]  (Australia) | Design: *RCT*  DHI type: *Website*  Participants: *Adults aged ≥18 years with a BMI of 25-40 kg/m^2^, who engaged in <150 minutes per week of MVPA*  Total N: *243*  Outcome: *Use, User experience* | Description: *A web-based tailored physical activity program focused on enhancing intrinsic motivation, self-efficacy, intentions for increasing activity levels, and self-regulatory strategies with or without activity trackers*  Conditions:  *Arm 1: Website plus activity tracker*  *Arm 2: Website*  Duration: *3 months* |
| Walthouwer, 2015 [87, 122]  (Netherlands) | Design: *RCT*  DHI type: *Website*  Participants: *Adults aged ≥18 years old with a BMI of 18.5-30 kg/m^2^*  Total N: *956*  Outcome: *Use, User experience* | Description: *A web-based tailored weight management program with information provided via text or videos*  Conditions:  *Arm 1:* *Website with mostly video education*  *Arm 2: Website with text only education*  Duration: *3 months* |
| Wang,  2015 [88, 113]  (United States) | Design: *RCT*  DHI type: *Activity tracker*  Participants: *Overweight or obese non-smokers aged 18 to 69 years, engaging in <150 minutes of MVPA per week*  Total N: *67*  Outcome: *Use* | Description: *A wearable physical activity monitoring device (Fitbit) with or without daily SMS text message physical activity prompts*  Conditions:  Arm 1: *Activity tracker plus SMS text message prompts*  Arm 2: *Activity tracker*  Duration: *6 weeks* |
| Webber,  2010 [33]  (United States) | Design: *RCT*  DHI type: *Website*  Participants: *Women aged 22 to 65 years with a BMI of 25-40 kg/m^2^*  Total N: *80*  Outcome: *Use* | Description: *A web-based behavioral weight loss program with or without MI style group sessions*  Conditions:  *Arm 1: Website plus motivation enhanced group sessions*  *Arm 2: Website plus standard group sessions*  Duration: *Up to 3 months* |
| West,  2016 [89]  (United States) | Design: *RCT*  DHI type: *Website*  Participants: *Adults aged ≥18 years, with a BMI of 25-50 kg/m^2^, in generally good health*  Total N: *398*  Outcome: *Use* | Description: *A web-based weight control program with or without group web-based MI chats*  Conditions:  *Arm 1: Website plus MI group sessions*  *Arm 2: Website only*  Duration: *18 months* |
| West,  2020 [55]  (United States) | Design: Cluster *RCT*  DHI type: *Website*  Participants: *Adults aged ≥18 years with a BMI of 25-50 kg/m^2^*  Total N: *418*  Outcome: *Use* | Description: *A web-based weight control program with or without financial incentives*  Conditions:  *Arm 1: Web-based weight control program plus financial incentives*  *Arm 2: Web-based weight control program*  Duration: *6 months* |
