# Supplementary material for: The Effectiveness of Strategies to Improve User Engagement With Digital Health Interventions Targeting Nutrition, Physical Activity, and Overweight and Obesity: Systematic Review and Meta-Analysis
Source: J Med Internet Res. 2023 Dec 19;25:e47987. doi: 10.2196/47987 (PMC10762625; doi:10.2196/47987)
Supplement: Multimedia Appendix 5 [file jmir_v25i1e47987_app5.docx]

**Multimedia Appendix 5. Risk of bias summary**

| **Study** | **Random sequence generation** | **Allocation concealment** | **Blinding of participants and personnel (use outcomes)** | **Blinding of participants and personnel (user experience outcomes)** | **Blinding of outcome assessment (use outcomes)** | **Blinding of outcome assessment (user experience outcomes)** | **Incomplete outcome data (use outcomes)** | **Incomplete outcome data (user experience outcomes)** | **Selective outcome reporting** | **Other bias (recruitment to cluster)** | **Other bias (baseline imbalance)** | **Other bias (loss of cluster)** | **Other bias (incorrect analysis)** | **Other bias (contamination)** | **Other bias (compatibility with individually randomized RCTs)** |
| --- | --- | --- | --- | --- | --- | --- | --- | --- | --- | --- | --- | --- | --- | --- | --- |
|  |  |  |  |  |  |  |  |  |  |  |  |  |  |  |  |
| Alley  2014 | L | L | U | U | L | L | L | L | L | NA | NA | NA | NA | NA | NA |
| NewtonJr 2014 | L | L | U | NA | L | NA | L | NA | U | NA | NA | NA | NA | NA | NA |
| Ross  2016 | L | U | H | NA | L | NA | L | NA | L | NA | NA | NA | NA | NA | NA |
| Soetens  2014 / Vandelanotte 2011 | L | L | U | U | L | H | H | H | L | NA | NA | NA | NA | NA | NA |
| Sze  2015 | U | L | L | L | L | H | L | L | L | NA | NA | NA | NA | NA | NA |
| Walthouwer 2015 | L | L | H | H | L | H | H | H | L | NA | NA | NA | NA | NA | NA |
| Wang  2015 | L | L | U | NA | H | NA | L | NA | U | NA | NA | NA | NA | NA | NA |
| Webber  2010 | U | U | U | NA | L | NA | L | NA | L | NA | NA | NA | NA | NA | NA |
| Vandelanotte 2017 | L | L | H | H | H | H | H | H | L | NA | NA | NA | NA | NA | NA |
| Pullen  2008 | U | U | U | NA | L | NA | H | NA | U | NA | NA | NA | NA | NA | NA |
| Fanning  2017 | H | H | L | NA | L | NA | L | NA | L | NA | NA | NA | NA | NA | NA |
| Mailey  2016 | L | U | U | NA | L | NA | L | NA | L | NA | NA | NA | NA | NA | NA |
| Brindal  2019 | L | U | L | L | L | L | H | H | L | NA | NA | NA | NA | NA | NA |
| Tsai  2007 | U | U | U | U | U | U | L | L | L | NA | NA | NA | NA | NA | NA |
| Shaw  2012 | L | U | U | U | U | U | H | H | L | NA | NA | NA | NA | NA | NA |
| Kleimann 2019 | L | L | H | NA | L | NA | H | NA | L | NA | NA | NA | NA | NA | NA |
| Monroe  2019 | L | L | H | H | L | H | L | L | L | NA | NA | NA | NA | NA | NA |
| Patel  2019 | L | L | H | NA | L | NA | L | NA | L | NA | NA | NA | NA | NA | NA |
| West  2016 | U | U | U | NA | U | NA | L | NA | H | NA | NA | NA | NA | NA | NA |
| Chai  2019 | L | L | U | U | L | U | H | H | L | NA | NA | NA | NA | NA | NA |
| Micco  2007 | U | U | U | NA | U | NA | U | NA | L | NA | NA | NA | NA | NA | NA |
| Ellingson 2019 | U | U | H | NA | U | NA | L | NA | L | NA | NA | NA | NA | NA | NA |
| Nour  2019 | L | L | L | NA | L | NA | L | NA | L | NA | NA | NA | NA | NA | NA |
| Nuijten  2019 | U | U | L | NA | L | NA | L | NA | L | L | L | L | L | U | U |
| Omran  2018 | L | L | U | NA | U | NA | L | NA | L | NA | NA | NA | NA | NA | NA |
| Kwan  2013 | L | L | L | NA | L | NA | H | NA | U | L | U | L | U | U | U |
| Liao  2020 | U | L | L | L | L | L | L | L | L | L | L | L | L | U | U |
| Guagliano 2019 | L | L | U | L | L | H | L | L | L | L | U | L | H | H | U |
| Vandelanotte 2018 | L | L | U | U | U | U | H | H | U | NA | NA | NA | NA | NA | NA |
| Edney  2019-2020 | L | L | U | NA | L | NA | L | NA | L | L | L | L | L | U | U |
| Alley  2016 | L | U | H | H | L | U | L | L | L | NA | NA | NA | NA | NA | NA |
| Kolt  2017 | L | U | U | U | L | U | L | L | L | NA | NA | NA | NA | NA | NA |
| Blanson-Henkemans 2009 | L | U | L | L | L | L | H | H | L | NA | NA | NA | NA | NA | NA |
| Brindal  2012 | H | U | L | L | L | L | L | L | L | NA | NA | NA | NA | NA | NA |
| Hutchesson 2016 / Collins 2012-2013 | L | L | L | NA | L | NA | L | NA | L | NA | NA | NA | NA | NA | NA |
| Couper  2010 | U | U | U | NA | L | NA | L | NA | L | NA | NA | NA | NA | NA | NA |
| Dennison 2014 | L | L | H | NA | L | NA | L | NA | L | NA | NA | NA | NA | NA | NA |
| Gabriele 2009 – 2011 | L | H | L | NA | L | NA | L | NA | L | NA | NA | NA | NA | NA | NA |
| LaRose  2019 | L | U | U | U | L | H | H | H | L | NA | NA | NA | NA | NA | NA |
| Napolitano 2013 | U | U | U | NA | L | NA | L | NA | L | NA | NA | NA | NA | NA | NA |
| Alley  2023 | L | L | U | U | L | U | L | U | L | NA | NA | NA | NA | NA | NA |
| Beleigoli  2020 | L | L | H | NA | L | NA | L | NA | L | NA | NA | NA | NA | NA | NA |
| Butryn  2020 | U | U | U | U | L | U | L | L | L | NA | NA | NA | NA | NA | NA |
| Eisenhauer  2021 | L | L | H | NA | L | NA | L | NA | L | NA | NA | NA | NA | NA | NA |
| Forman  2019 | L | H | NA | U | NA | U | NA | L | L | NA | NA | NA | NA | NA | NA |
| Granet  2023 | U | U | U | U | L | U | H | H | L | NA | NA | NA | NA | NA | NA |
| Haslam  2023 | L | L | NA | U | NA | U | NA | H | L | NA | NA | NA | NA | NA | NA |
| Jin  2021 | L | U | U | U | U | U | U | U | L | NA | NA | NA | NA | NA | NA |
| LaRose  2020(a) | U | U | U | U | L | U | L | H | U | NA | NA | NA | NA | NA | NA |
| LaRose  2020(b) / Leahey 2020 | U | U | U | NA | L | NA | L | NA | U | NA | NA | NA | NA | NA | NA |
| Levin  2022 | L | L | U | U | L | U | L | U | U | NA | NA | NA | NA | NA | NA |
| Pischke  2022 | U | U | H | H | H | H | H | H | L | NA | NA | NA | NA | NA | NA |
| Schoeppe  2022 / Vandelanotte 2021 | L | L | U | U | U | H | H | H | L | NA | NA | NA | NA | NA | NA |
| West  2020 | L | U | U | NA | L | NA | U | NA | L | L | L | U | L | U | U |

| **Key:** | |
| --- | --- |
| L | Low risk of bias |
| H | High risk of bias |
| U | Unclear risk of bias |
| N/A | Not applicable to this study |
|  |  |
