# Supplementary material for: The Effectiveness of Strategies to Improve User Engagement With Digital Health Interventions Targeting Nutrition, Physical Activity, and Overweight and Obesity: Systematic Review and Meta-Analysis
Source: J Med Internet Res. 2023 Dec 19;25:e47987. doi: 10.2196/47987 (PMC10762625; doi:10.2196/47987)
Supplement: Multimedia Appendix 6 [file jmir_v25i1e47987_app6.docx]

**Multimedia Appendix 6. Excluded studies and outcomes**

**Table S1 Excluded studies and outcomes from the use meta-analysis and meta-regression**

| **Author (year)** | **Outcome** | **Reason** |
| --- | --- | --- |
| Pullen (2008) | Times read newsletters | No measure of variability |
| LaRose (2019) | Calories reported via web | No measure of variability |
| Alley (2016) | Number of website visits | No measure of variability |
| Napolitano (2013) | Number of likes (average) | No measure of variability |
| Napolitano (2013) | Number of RSVPs (average) | No measure of variability |
| Napolitano (2013) | Number of posts (average) | No measure of variability |
| Nuijten (2019)^a^ | Number of days online (change) | Measured difference in the rate of change throughout the intervention, rather than difference at follow-up |
| Nuijten (2019)^a^ | Number of activities (change) | Measured difference in the rate of change throughout the intervention, rather than difference at follow-up |
| Tsai (2007) | Entered calorie consumption | No measure of variability |
| Tsai (2007) | Entry of caloric expenditure | No measure of variability |
| Edney (2019-2020) | Superuser (top quartile of users) | No measure of variability |
| Vandelanotte (2017) | Number using the website | No measure of variability |
| Forman (2019)^a^ | NA | No use outcomes |
| Haslam (2023)^a^ | NA | No use outcomes |
| Eisenhauer (2021) | Weeks monitored diet | No measure of variability |
| Eisenhauer (2021) | Weeks monitored weight | No measure of variability |

^a^ Study excluded from meta-analysis and meta-regressions.

**Table S2 Excluded studies and outcomes from the user experience meta-analysis and meta-regression**

| **Author (year)** | **Outcome** | **Reason** |
| --- | --- | --- |
| Kolt (2017)^a^ | Systems usability score | Insufficient data reported to allow for an SMD to be calculated |
| Alley (2016)^a^ | Program satisfaction score | Insufficient data reported to allow for an SMD to be calculated |
| Monroe (2019)^a^ | Satisfied with program | Insufficient data reported to allow for an SMD to be calculated |
| Monroe (2019)^a^ | Expanded social network | Insufficient data reported to allow for an SMD to be calculated |
| LaRose (a) (2020)^a^ | Recommend to a friend | No measure of variance |
| Schoeppe (2022)/ Vandelanotte (2021) | System usability score (SUS) | No measure of variance |
| Pullen (2008)^a^ | NA | No user experience outcomes |
| Patel (2019)^a^ | NA | No user experience outcomes |
| Edney (2019-2020)^a^ | NA | No user experience outcomes |
| Ellingson (2019)^a^ | NA | No user experience outcomes |
| Fanning (2017)^a^ | NA | No user experience outcomes |
| Kwan (2013)^a^ | NA | No user experience outcomes |
| Mailey (2016)^a^ | NA | No user experience outcomes |
| Newton (2014)^a^ | NA | No user experience outcomes |
| Omran (2018)^a^ | NA | No user experience outcomes |
| Wang (2015)^a^ | NA | No user experience outcomes |
| Dennison (2014)^a^ | NA | No user experience outcomes |
| Gabriele (2009-2011)^a^ | NA | No user experience outcomes |
| Hutchesson (2016)/ Collins (2012-2013)^a^ | NA | No user experience outcomes |
| Kleimann (2019)^a^ | NA | No user experience outcomes |
| Micco (2007)^a^ | NA | No user experience outcomes |
| Napolitano (2013)^a^ | NA | No user experience outcomes |
| Nuijten (2019)^a^ | NA | No user experience outcomes |
| Ross (2016)^a^ | NA | No user experience outcomes |
| Webber (2010)^a^ | NA | No user experience outcomes |
| West (2016)^a^ | NA | No user experience outcomes |
| Couper (2010)^a^ | NA | No user experience outcomes |
| Nour (2019)^a^ | NA | No user experience outcomes |
| Beleigoli (2020)^a^ | NA | No user experience outcomes |
| Eisenhauer (2021)^a^ | NA | No user experience outcomes |
| LaRose (b) (2020)/ Leahey (2020)^a^ | NA | No user experience outcomes |
| West (2020)^a^ | NA | No user experience outcomes |

^a^ Study excluded from meta-analysis and meta-regressions.
