# Supplementary material for: The Effectiveness of Strategies to Improve User Engagement With Digital Health Interventions Targeting Nutrition, Physical Activity, and Overweight and Obesity: Systematic Review and Meta-Analysis
Source: J Med Internet Res. 2023 Dec 19;25:e47987. doi: 10.2196/47987 (PMC10762625; doi:10.2196/47987)

**Multimedia Appendix 7. Meta-Analysis and Meta-Regression Results**

**1. Use outcomes meta-analysis and meta-regression**

Table S1 Robust multivariate meta-analysis

|  | Effect estimate | SE | *P*-value | 95% CI | I^2^ | GRADE rating |
| --- | --- | --- | --- | --- | --- | --- |
| SMD | 0.329 | 0.063 | <0.001 | 0.202 - 0.456 | 85.90 | Very low^a^ |

^a^ Certainty of evidence downgraded one level for inconsistency (substantial heterogeneity I^2^= 85.9%); downgraded one level for indirectness; downgraded one level for imprecision

Table S2 Robust multivariate meta-analysis- sensitivity (excluding medians)

|  | Effect estimate | SE | *P*-value | 95% CI | I^2^ |
| --- | --- | --- | --- | --- | --- |
| SMD | 0.306 | 0.062 | <0.001 | 0.181 - 0.431 | 84.4 |

Table S3 Sensitivity results - varying the within-study effect size correlation (rho)

|  |  | **Rho = 0** | **Rho = 0.2** | **Rho = 0.4** | **Rho = 0.6** | **Rho = 0.8** | **Rho = 1** |
| --- | --- | --- | --- | --- | --- | --- | --- |
| SMD | Coefficient | 0.3291 | 0.3291 | 0.3291 | 0.3292 | 0.3292 | 0.3292 |
|  | SE | 0.0633 | 0.0633 | 0.0633 | 0.0633 | 0.0633 | 0.0634 |
| Tau.sq | Estimate | 0.1420 | 0.1420 | 0.1421 | 0.1421 | 0.1422 | 0.1422 |

Table S4 Robust multivariate meta-analysis- sensitivity (excluding outlier Alley 2014)

|  | Effect estimate | SE | *P*-value | 95% CI | I^2^ |
| --- | --- | --- | --- | --- | --- |
| SMD | 0.308 | 0.059 | <0.001 | 0.189 - 0.427 | 83.1 |

Table S5 Robust multivariate meta-analysis – sensitivity (excluding studies at high risk of bias)

|  | Effect estimate | SE | *P*-value | 95% CI | I^2^ |
| --- | --- | --- | --- | --- | --- |
| SMD | 0.359 | 0.066 | <0.001 | 0.227 - 0.492 | 85.0 |

Table S6 Robust multivariate regression- study type (Overweight and Obesity, Physical Activity (PA), Nutrition studies)

|  | Effect estimate | SE | *P*-value | 95% CI | I^2^ |
| --- | --- | --- | --- | --- | --- |
| Intercept | 0.31 | 0.095 | 0.002 | 0.121 - 0.50 | 85.32 |
| Nutrition (vs Overweight and Obesity) | -0.167 | 0.149 | 0.27 | -0.467 - 0.133 | 85.32 |
| PA (vs Overweight and Obesity) | 0.067 | 0.138 | 0.63 | -0.212 - 0.345 | 85.32 |

Table S7 Robust meta-regression-technology type

|  | Effect estimate | SE | *P*-value | 95% CI | I^2^ |
| --- | --- | --- | --- | --- | --- |
| Technology - website | 0.038 | 0.15 | 0.81 | -0.270 - 0.346 | 85.82 |
| Technology - mobile apps | 0.202 | 0.196 | 0.31 | -0.192 - 0.596 | 85.64 |
| Technology - activity tracker | 0.085 | 0.337 | 0.80 | -0.591 - 0.761 | 85.86 |

Table S8 Robust meta-regression-engagement measure type

|  | Effect estimate | SE | *P*-value | 95% CI | I^2^ |
| --- | --- | --- | --- | --- | --- |
| Intercept | 0.338 | 0.078 | <0.01 | 0.181 - 0.495 | 85.83 |
| Time spent | -0.115 | 0.129 | 0.38 | -0.375 - 0.145 | 85.83 |
| Log-ins | 0.013 | 0.107 | 0.91 | -0.202 - 0.228 | 85.83 |

Table S9 Robust meta-regression-study setting (closed, personal, tightly controlled vs open remote data collection and delivery)

|  | Effect estimate | SE | *P*-value | 95% CI | I^2^ |
| --- | --- | --- | --- | --- | --- |
| Intercept | 0.425 | 0.112 | <0.001 | 0.200 - 0.65 | 84.8 |
| Closed vs open remote | -0.187 | 0.128 | 0.15 | -0.443 - 0.07 | 84.8 |

Table S10 Robust meta-regression: multivariate regression of differences between arms

| Meta-regression | Effect Estimate | SE | *P*-value | 95% CI | I^2^ |
| --- | --- | --- | --- | --- | --- |
| BCT- social support | 0.40 | 0.13 | <0.001 | 0.14 - 0.66 | 85.19 |
| BCT – reward and threat | 0.21 | 0.14 | 0.14 | -0.07 - 0.48 | 85.48 |
| BCT - antecedents | 0.12 | 0.29 | 0.68 | -0.46 - 0.70 | 85.78 |
| BCT - associations | 0.19 | 0.24 | 0.43 | -0.29 - 0.67 | 84.86 |
| BCT – feedback and monitoring | 0.33 | 0.27 | 0.24 | -0.22 - 0.88 | 85.67 |
| BCT – goals and planning | 0.33 | 0.23 | 0.15 | -0.13 - 0.78 | 85.23 |
| BCT – scheduled consequences | 0.19 | 0.13 | 0.15 | -0.07 - 0.44 | 85.62 |
| BCT – repetition and substitution | 0.51 | 0.30 | 0.10 | -0.10 - 1.12 | 85.07 |
| BCT – shaping knowledge | 0.39 | 0.18 | 0.03 | 0.03 - 0.74 | 85.40 |
| Design feature – (a) enriched information environment | 0.29 | 0.20 | 0.15 | -0.11 - 0.69 | 85.85 |
| Design feature – (b) automated tailored feedback | 0.29 | 0.17 | 0.09 | -0.05 – 0.63 | 85.46 |
| Design feature – (c) automated follow-up messages | 0.03 | 0.27 | 0.90 | -0.51- 0.58 | 85.29 |
| Design feature – (f) peer-to-peer access | 0.14 | 0.11 | 0.22 | -0.09 – 0.37 | 85.85 |
| Design feature - (g) email | 0.39 | 0.21 | 0.07 | -0.04 - 0.81 | 85.04 |
| Design feature - (h) telephone | 0.41 | 0.25 | 0.11 | -0.10 – 0.92 | 85.53 |

Figure S1 Use outcomes meta-analysis forest plot


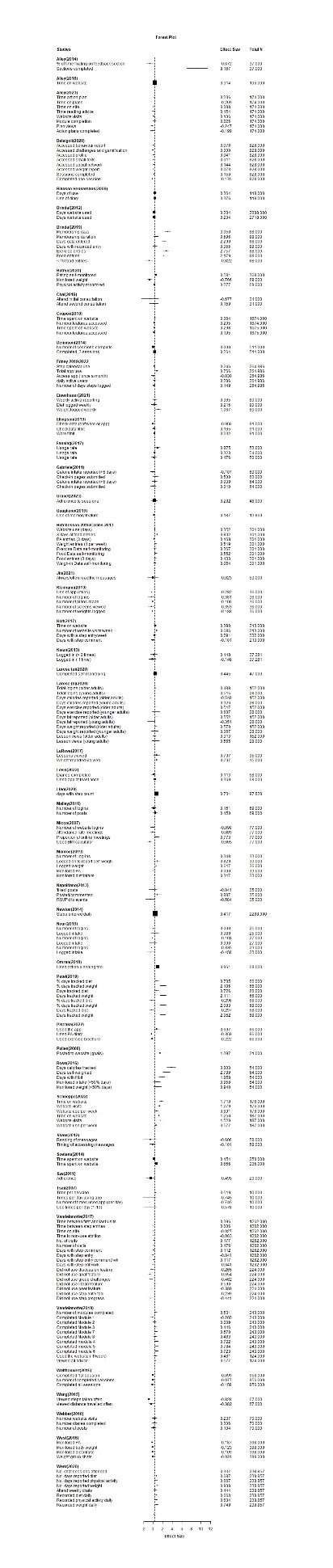


**2. User experience outcomes meta-analysis and meta-regression**

Table S11 Robust multivariate meta-analysis

|  | Effect estimate | SE | *P*-value | 95% CI | I^2^ | GRADE rating |
| --- | --- | --- | --- | --- | --- | --- |
| SMD | 0.294 | 0.107 | 0.01 | 0.072 - 0.516 | 78.0 | Very low^a^ |

^a^ Certainty of evidence down-graded one level for inconsistency (substantial heterogeneity I^2^= 82.9%); downgraded one level for indirectness; downgraded one level for imprecision.

Table S12 Sensitivity results - varying the within-study effect size correlation (rho)

|  |  | **Rho = 0** | **Rho = 0.2** | **Rho = 0.4** | **Rho = 0.6** | **Rho = 0.8** | **Rho = 1** |
| --- | --- | --- | --- | --- | --- | --- | --- |
| SMD | Coefficient | 0.294 | 0.294 | 0.29 | 0.294 | 0.294 | 0.294 |
|  | SE | 0.107 | 0.107 | 0.11 | 0.107 | 0.107 | 0.107 |
| Tau.sq | Estimate | 0.152 | 0.153 | 0.15 | 0.153 | 0.153 | 0.153 |

Table S13 Robust multivariate meta-analysis – sensitivity (excluding studies at high risk of bias)

|  | Effect estimate | SE | *P*-value | 95% CI | I^2^ |
| --- | --- | --- | --- | --- | --- |
| SMD | 0.339 | 0.124 | 0.01 | 0.081 - 0.597 | 79.4 |

Table S14 Robust meta-regression: multivariate regression of differences between arms

| Meta-regression | Effect Estimate | SE | *P*-value | 95% CI | I^2^ |
| --- | --- | --- | --- | --- | --- |
| BCT - social support | 0.70 | 0.25 | 0.01 | 0.18 - 1.22 | 68.63 |
| BCT – feedback and monitoring | 0.18 | 0.14 | 0.22 | -0.11 - 0.47 | 78.07 |
| BCT – self belief | 0.11 | 0.20 | 0.60 | -0.31 - 0.52 | 78.49 |
| BCT – reward and threat | 0.59 | 0.45 | 0.20 | -0.34 - 1.53 | 73.55 |
| BCT – goals and planning | 0.17 | 0.15 | 0.27 | -0.14 - 0.48 | 78.35 |
| BCT - associations | 0.17 | 0.15 | 0.27 | -0.14 - 0.48 | 78.35 |
| BCT – repetition and substitution | 0.29 | 0.11 | 0.02 | 0.05 - 0.53 | 78.33 |
| BCT – natural consequences | 0.29 | 0.11 | 0.02 | 0.05 - 0.53 | 78.33 |
| Design feature - (a) enriched information environment | 0.13 | 0.20 | 0.53 | -0.29 – 0.54 | 78.76 |
| Design feature - (b) automated tailored feedback | 0.00 | 0.20 | 0.99 | -0.41 - 0.41 | 78.87 |
| Design feature – (c) automated follow-up messages | 0.24 | 0.17 | 0.18 | -0.12 – 0.60 | 78.41 |
| Design feature - (g) email | 0.29 | 0.11 | 0.02 | 0.05 - 0.53 | 78.33 |
| Design feature - (h) telephone contacts | -0.01 | 0.30 | 0.96 | -0.64 - 0.62 | 78.65 |
| Design feature – (i) SMS text messages | 0.34 | 0.11 | 0.01 | 0.11 – 0.57 | 78.75 |

Table S15 Robust multivariate regression- study type (Physical Activity (PA), Overweight and Obesity, Nutrition studies)

|  | Effect estimate | SE | *P*-value | 95% CI | I^2^ |
| --- | --- | --- | --- | --- | --- |
| Intercept | 0.167 | 0.116 | 0.16 | -0.074 – 0.408 | 89.449 |
| PA (vs Overweight and Obesity) | 0.272 | 0.234 | 0.26 | -0.214 – 0.758 | 89.449 |
| Diet (vs Overweight and Obesity) | 0.189 | 0.116 | 0.112 | -0.051 – 0.430 | 79.449 |

Table S16 Robust meta-regression-technology type

|  | Effect estimate | SE | *P*-value | 95% CI | I^2^ |
| --- | --- | --- | --- | --- | --- |
| Technology - website | 0.140 | 0.268 | 0.61 | -0.415 – 0.695 | 78.88 |
| Technology- mobile apps | -0.463 | 0.176 | 0.02 | -0.829 - -0.097 | 77.70 |
| Technology - telehealth | 0.044 | 0.160 | 0.79 | -0.288 – 0.375 | 78.84 |
| Technology – SMS text messages | -0.433 | 0.142 | 0.01 | -0.728 - -0.139 | 78.62 |

Table S17 Robust meta-regression-study setting (closed, personal, tightly controlled vs open remote data collection and delivery)

|  | Effect estimate | SE | *P*-value | 95% CI | I^2^ |
| --- | --- | --- | --- | --- | --- |
| Intercept | 0.428 | 0.228 | 0.07 | -0.044 - 0.901 | 77.0 |
| Closed vs open remote | -0.225 | 0.245 | 0.37 | -0732 - 0.282 | 77.0 |

Figure S2 User experience meta-analysis forest plot


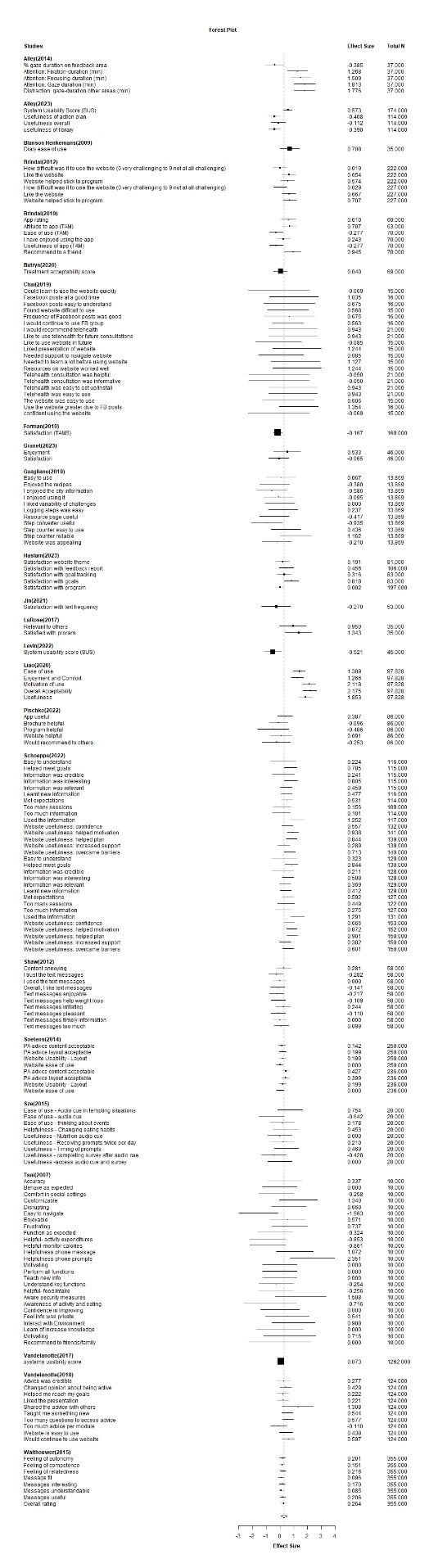

Supplement: Multimedia Appendix 7 [file jmir_v25i1e47987_app7.docx]
